# The dynein adaptor Hook2 plays essential roles in mitotic progression and cytokinesis

**DOI:** 10.1083/jcb.201804183

**Published:** 2019-03-04

**Authors:** Devashish Dwivedi, Amrita Kumari, Siddhi Rathi, Sivaram V.S. Mylavarapu, Mahak Sharma

**Affiliations:** 1 Department of Biological Sciences, Indian Institute of Science Education and Research, Mohali, India; 2 Laboratory of Cellular Dynamics, Regional Centre for Biotechnology, Faridabad, India; 3 Affiliated to Manipal Academy of Higher Education, Manipal, India

## Abstract

Hook proteins are evolutionarily conserved dynein adaptors that promote assembly of highly processive dynein–dynactin motor complexes. Mammals express three Hook paralogs, namely Hook1, Hook2, and Hook3, that have distinct subcellular localizations and expectedly, distinct cellular functions. Here we demonstrate that Hook2 binds to and promotes dynein–dynactin assembly specifically during mitosis. During the late G2 phase, Hook2 mediates dynein–dynactin localization at the nuclear envelope (NE), which is required for centrosome anchoring to the NE. Independent of its binding to dynein, Hook2 regulates microtubule nucleation at the centrosome; accordingly, Hook2-depleted cells have reduced astral microtubules and spindle positioning defects. Besides the centrosome, Hook2 localizes to and recruits dynactin and dynein to the central spindle. Dynactin-dependent targeting of centralspindlin complex to the midzone is abrogated upon Hook2 depletion; accordingly, Hook2 depletion results in cytokinesis failure. We find that the zebrafish Hook2 homologue promotes dynein–dynactin association and was essential for zebrafish early development. Together, these results suggest that Hook2 mediates assembly of the dynein–dynactin complex and regulates mitotic progression and cytokinesis.

## Abstract (precis)

Hook proteins are conserved dynein adaptors that promote the assembly of dynein–dynactin motor complexes. Dwivedi et al. show that Hook2 is a novel mitotic adaptor that mediates dynein–dynactin–dependent anchoring of the centrosome to the nuclear envelope and microtubule nucleation from mitotic centrosomes. In addition, Hook2 is needed for the dynactin-dependent targeting of the centralspindlin complex to the midzone for normal cytokinesis.

## Introduction

Cytoplasmic dynein 1 (hereafter referred to as “dynein”) is a large microtubule (MT)-based motor protein that mediates long-range retrograde transport of organelles, endosomes, proteins, and RNA granules toward the minus ends of MTs. Dynein also has multiple functions during cell division, including centrosome separation and nuclear envelope (NE) breakdown (NEBD), chromosome alignment, spindle pole focusing, spindle orientation and positioning, and spindle assembly checkpoint inactivation (Sharp et al., 2000; Howell et al., 2001; Salina et al., 2002; Goshima et al., 2005; Varma et al., 2008; Raaijmakers et al., 2012, 2013). Dynein is a homodimer of two heavy chain subunits that bind and hydrolyze ATP, and act as a scaffold to form a complex with two intermediate chains, two light intermediate chains (LICs), and homodimers of three light chains (LL1/2, Roadblock-1/2, and TCTex1/1L; Pfister et al., 2005, 2006; Kardon and Vale, 2009). On its own, mammalian dynein is not a processive motor; rather, association with the multisubunit dynactin complex and the coiled-coil activating adaptor proteins is required for dynein processive motility (Trokter et al., 2012; McKenney et al., 2014; Schlager et al., 2014; Lee et al., 2018). The coiled-coil activating adaptors including Bicaudal D2 (BICD2), Rab11-FIP3, and Spindly share the ability to interact with both dynein and dynactin to promote dynein processive motility, and also regulate dynein–dynactin recruitment on the cargo surface (Griffis et al., 2007; Horgan et al., 2010; Splinter et al., 2012; McKenney et al., 2014; Schlager et al., 2014).

**Figure 1.**
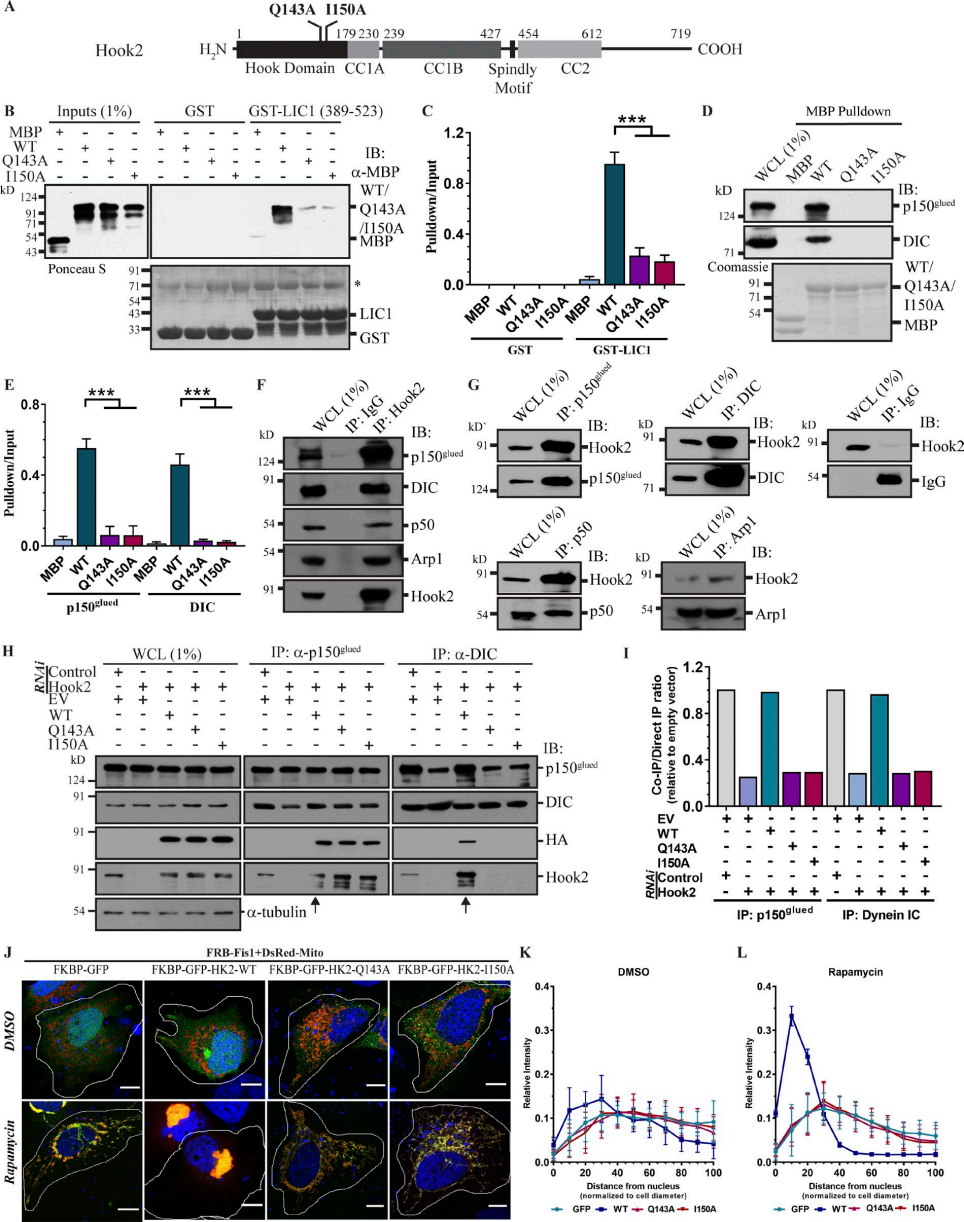
**Hook2 acts as a dynein–dynactin linker. (A)** Domain architecture of Hook2 and its domain deletion fragments/mutants used in the study. **(B)** GST or GST-tagged LIC1 (389–523 aa) bound to glutathione beads were incubated with MBP-tagged Hook2 N427 (WT, Q143A, and I150A), and immunoblotted (IB) with an anti-MBP antibody for Hook2 (WT/mutants). LIC1 in the pelleted beads was detected using Ponceau S staining of the membrane. The asterisk indicates BSA protein band used for blocking glutathione beads. **(C)** Ratio of band intensity of pulldown to input Hook2 fragment signals in B (*n* = 3). **(D)** HEK293T cell lysates were incubated with MBP alone or MBP-tagged Hook2 N427 (WT, Q143A, and I150A) bound to amylose beads, and IB for DIC and p150^glued^. The amount of recombinant Hook2 (WT/mutants) protein was analyzed by Coomassie staining. **(E)** Ratio of band intensity of pulldown to input Hook2 (WT/mutants) signal in D (*n* = 3). **(F)** Protein-A/G beads bound to control IgG or anti-Hook2 antibody were incubated with HEK293T lysates; the interactome IP was IB to check the presence of different dynein subunits. **(G)** Protein-A/G beads bound to antibodies against DIC, p150^glued^, Arp1, and p50/dynamitin were incubated with HEK293T lysate; the interactome IP was IB to check the presence of Hook2. **(H)** Lysates from HEK293T cells treated with control or Hook2 siRNA and transfected with indicated plasmids were incubated with protein-G beads bound to antibodies against DIC and p150^glued^, and IP were IB with the indicated antibodies. Arrows mark Hook2 (WT) transfected lanes. **(I)** Ratio of normalized band intensity (EV) of IP DIC to p150^glued^ and vice versa in H (*n* = 2). **(J)** Representative images of FRB-FKBP12-rapamycin dimerization assay in fixed HeLa cells. Bars, 10 µm. **(K and L)** Mitochondrial distribution quantified as intensity with respect to relative distance from the nucleus (*n* = 3; 10 cells/experiment). Data represent mean ± SD (***, P < 0.001; Student’s *t* test).

Recent studies have characterized a novel family of evolutionarily conserved dynein adaptors (“Hook proteins”) that contain an N-terminal Hook domain, two central coiled-coil domains, and a C-terminal organelle binding region (Walenta et al., 2001; McKenney et al., 2014; Olenick et al., 2016; Fig. 1 A). Hook orthologues in fungi and worms bind dynein via their Hook superfamily domain (Malone et al., 2003; Bielska et al., 2014; Zhang et al., 2014). Fungal Hook protein, HookA, promotes dynein recruitment to the early endosomes, mediating their retrograde motility (Bielska et al., 2014; Zhang et al., 2014). Unlike fungi, flies, and worms where a single Hook protein is present, mammals have three Hook paralogs, namely, Hook1, Hook2, and Hook3, that exhibit a high degree of sequence conservation in the N-terminal Hook domain and a divergent sequence in the C-terminal region (Krämer and Phistry, 1999; Walenta et al., 2001).

Several recent studies have directly investigated the dynein–dynactin activating adaptor function of human Hook1 and Hook3 proteins (McKenney et al., 2014; Olenick et al., 2016; Schroeder and Vale, 2016; Redwine et al., 2017; Grotjahn et al., 2018; Lee et al., 2018; Urnavicius et al., 2018). Hook3, like BICD2, Spindly, and Rab11-FIP3, forms a stable ternary complex with dynein and dynactin and promotes processive motility of the dynein–dynactin complex on the MT tracks (McKenney et al., 2014). Hook1 and Hook3 associate with the dynein–dynactin complex via direct binding of the Hook domain with the LIC1 subunit of dynein (Schroeder and Vale, 2016; Lee et al., 2018). Recent cryo-EM reconstruction studies of the dynein–dynactin–Hook3 complex have revealed that Hook3-bound dynactin primarily recruits two dynein molecules, which increases both the force and speed of the MT motor (Grotjahn et al., 2018; Urnavicius et al., 2018). Consistent with their role in dynein activation, Hook1 and Hook3 regulate retrograde motility of Rab5-positive axonal carriers (Guo et al., 2016) and of TrkB-BDNF–signaling endosomes (Hook1) in neurons (Olenick et al., 2018).

Unlike Hook1 and Hook3, little is known about Hook2 function as a dynein–dynactin activating adaptor. Here, we show that Hook2 is required for the assembly of the dynein–dynactin complex, and forced recruitment of Hook2 on organelle membranes is sufficient for their rapid transport in a dynein-dependent manner. Depletion of Hook2, but not other Hook paralogs, impaired dynein–dynactin association during prometaphase and early anaphase stages of the cell cycle. During the G2/M transition, Hook2 mediates centrosome anchoring to the NE, possibly by regulating CENP-F–mediated dynein–dynactin recruitment to the NE. Live-cell imaging revealed a delay in chromosome congression and spindle positioning defects in Hook2-depleted cells, which is likely due to Hook2 function in regulating MT nucleation at the centrosome. Despite these early defects, Hook2-depleted cells progressed to late anaphase but showed an incomplete cleavage furrow ingression, leading to the formation of binucleated cells. We found that Hook2 promotes dynactin and dynein localization to the central spindles; consequently, dynactin-dependent targeting of centralspindlin complex to the midzone is abrogated upon Hook2 depletion. The zebrafish Hook2 homologue localized to the centrosomes, recruited dynein–dynactin subunits to the centrosome, and acted as a linker to promote dynein–dynactin interaction, supporting an evolutionarily conserved function for Hook2. Taken together, our findings suggest that Hook2 promotes assembly of the dynein–dynactin complex during mitosis and regulates multiple stages of cell cycle progression and cytokinesis.

## Results

### The Hook domain of Hook2 binds to LIC1

Previous studies have shown that Hook3, via its N-terminal Hook domain (1–180 aa), binds to the dynein subunit LIC1 (Schroeder and Vale, 2016; Lee et al., 2018). Further Hook3 fragments encompassing both the Hook domain and coiled-coil domains (i.e., 1–239 aa and 1–434 aa) showed stronger binding to LIC1 (Schroeder and Vale, 2016). As the Hook domain sequence is highly conserved among the three Hook paralogs (∼60% sequence similarity and ∼47% sequence identity of Hook domain of Hook2 with Hook1 and Hook3; Fig. S1 A), we investigated whether Hook2, like Hook3, directly binds to LIC1. To this end, an equal amount of maltose-binding protein (MBP)–tagged versions of Hook2-Hook (N179), Hook+CC1A (N230), and Hook+CC1 (N427) proteins was incubated with either GST or GST-LIC1 (389–523), and interactions obtained after pulldown were analyzed by immunoblotting (Fig. S1, B and C). Indeed, all Hook2 fragments associated with GST-LIC1 (389–523) but not with GST (Fig. S1 B). Greater amounts of Hook2 (N230) and Hook2 (N427) were pulled down with GST-LIC1 (389–523) as compared with Hook2 (N179; Fig. S1, B and C), indicating that the coiled-coil domains strengthened binding to LIC1. Thus, similar to the other cargo-specific adaptors (Schroeder et al., 2014; Schroeder and Vale, 2016), Hook2 also directly associates with the adaptor-binding C-terminal region of LIC1. Two conserved residues (Q147 and I154) within the Hook domain of Hook3 were reported to be crucial for its interaction with LIC1 (Schroeder and Vale, 2016). Expectedly, similar point mutations in MBP-tagged Hook2 N427 fragment (Q143A and I150A, boxed in Fig. S1 A) abrogated binding to LIC1 (Fig. 1, B and C). Consistent with these findings, MBP-tagged Hook2 (N427) point mutants (Q143A and I150A) failed to pull down endogenous dynein and dynactin from HEK293T cell lysates, as compared with the WT protein (Fig. 1, D and E). These findings suggest that similar to Hook3, binding of Hook2 (N427) fragment to LIC1 is required for its association with the dynein–dynactin complex.

Next, we corroborated Hook2 interaction with dynein–dynactin subunits by coimmunoprecipitation (coIP) of the endogenous proteins. To this end, we first confirmed the specificity of anti-Hook antibodies by depleting Hook1, Hook2, or Hook3, using siRNA oligo sequences targeting particular Hook paralogs (Fig. S1 D). We observed coIP of the dynein subunit DIC and dynactin subunits p150^glued^, p50, and Arp1, with Hook2 and vice versa (Fig. 1, F and G). Taken together, our results suggest that Hook2 interacts with both dynein and dynactin under physiological conditions.

### Hook2 is a linker required for assembly of dynein–dynactin complex

We next investigated whether Hook2, similar to Hook3, acts as a linker between dynein and dynactin. We noted that Hook2 promotes interaction between dynein and dynactin, as overexpression of Hook2 WT, as compared with empty vector (EV), increased coIP of endogenous dynein with dynactin, and the reverse was also true (Fig. S1 E; compare EV lane with WT and Fig. S1 F). Surprisingly, the dynein–dynactin interaction was dramatically reduced upon overexpression of LIC binding-defective Hook2 point mutants (Q143A and I150A), suggesting that these mutants exert a dominant-negative effect on dynein–dynactin interaction (Fig. S1 E; compare WT lane with Q143A and I150A and Fig. S1 F). Upon probing for immunoprecipitated (IP) HA-tagged Hook2 in these experiments, we observed that Hook2 point mutants (Q143A and I150A) did not interact with dynein; however, they continued to interact with dynactin with similar binding as the WT (Fig. S1, E and F). Taking our observations of Fig. 1 B into consideration where the same point mutations disrupted binding to dynactin in the context of the Hook2 N427 fragment, this suggests that full-length Hook2 protein has an additional binding site(s) for dynactin that lie downstream of the CC1 region. Our results also indicate that the LIC binding-defective mutants of Hook2 function as dominant-negative by sequestering dynactin but not dynein and thereby disabling interaction with dynein.

Next, we used a siRNA-based approach to corroborate function of Hook2 as an assembly factor for the dynein–dynactin complex. Hook1 and Hook3 protein levels remained unaltered in Hook2 siRNA-treated cells, while levels of Hook2 were reduced by >90% (Fig. S1 D). Consistent with the overexpression data, depletion of Hook2 considerably reduced dynactin coIP with dynein and vice versa (Fig. 1, H and I). The specificity of Hook2 siRNA treatment was confirmed by rescue of dynein–dynactin interaction upon reintroduction of the WT version (siRNA-resistant Hook2 WT; Fig. 1, H and I). In line with our earlier results, we did not observe rescue of dynein–dynactin interaction upon expression of the LIC binding-defective point mutants (siRNA-resistant Hook2 Q143A/I150A; Fig. 1, H and I). Here also, we noted that LIC binding-defective mutations disrupt Hook2 interaction with DIC, but not with p150^glued^ (compare anti-HA lanes in IP eluates of both antibodies; Fig. 1, H and I). These findings indicate that Hook2 is required for dynein–dynactin interaction under physiological conditions.

Next, we tested whether Hook2, like other hook paralogs, is sufficient to recruit dynein to a target compartment/organelle and induce their retrograde motility. To this end, we used the FRB-FKBP-rapamycin–induced heterodimerization (Muthuswamy et al., 1999) wherein Hook2 was fused to the FKBP12 protein and, upon addition of rapamycin, the FKBP12-Hook2 fusion protein was rapidly translocated to the mitochondria where FRB-tagged FIS1 (mitochondrial protein) was localized (Fig. 1 J and Fig. S1 G). We confirmed that GFP-FKBP12-Hook2 retained its centrosomal localization in the absence of rapamycin (Fig. 1 J, as labeled). As shown in Fig. 1 J, GFP-FKBP12-Hook2 was recruited to mitochondria (labeled by DsRed-Mito) upon addition of rapamycin, and this was sufficient to induce tight clustering of mitochondria in the perinuclear region (Fig. 1, J–L). Hook2-dependent mitochondrial redistribution was dynein-dependent, as perinuclear clustering was not observed in dynein siRNA-treated cells (Fig. S1, H and I). Supporting this observation, the dynein binding-defective mutants of Hook2 (Q143A and I150A) failed to induce mitochondrial redistribution toward the cell center (Fig. 1, J–L), although these mutants were also similarly localized to the mitochondria as Hook2 WT (Fig. S1 G). Taken together, these findings suggest that Hook2 binding to dynein–dynactin results in a functional complex, which can support organelle motility.

### Hook2 is required for dynein–dynactin association upon entry into mitosis

**Figure 2.**
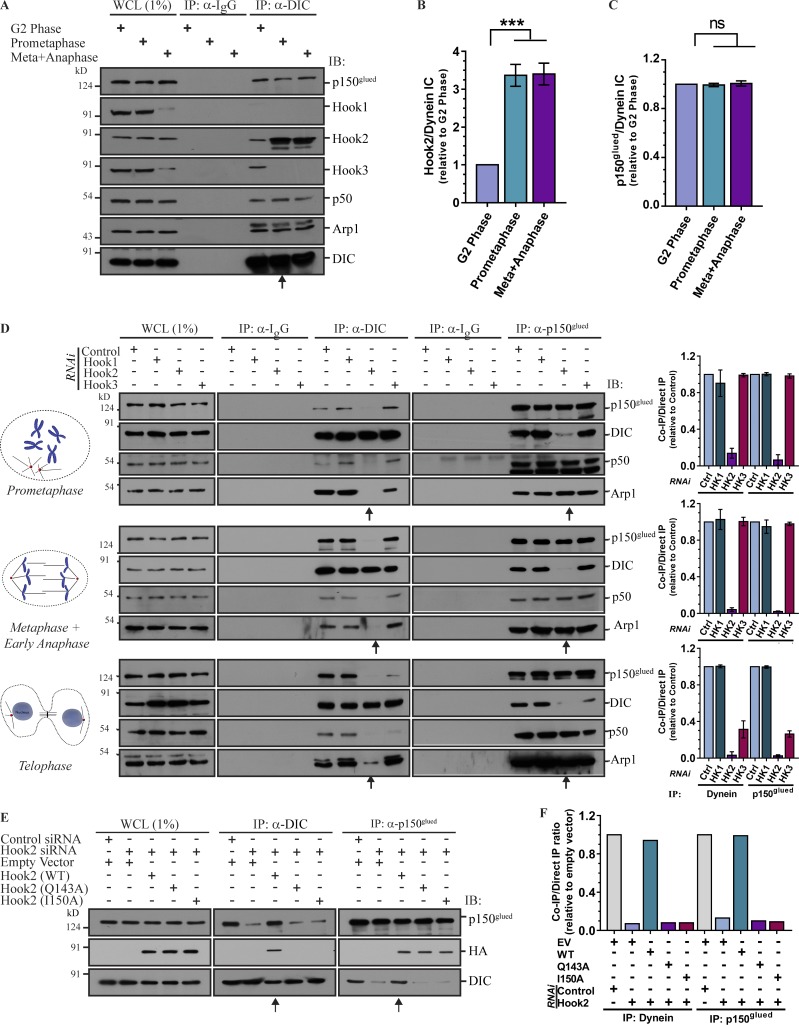
**Hook2 is required for dynein association with dynactin during mitosis. (A)** Lysates from HEK293T cells synchronized in G2 phase, prometaphase, and metaphase/anaphase were IP with control IgG or anti-DIC antibodies. The precipitates were IB with the indicated antibodies. Arrow mark prometaphase lane indicating an increased association of Hook2 with the dynein–dynactin complex at mitosis onset. **(B)** Ratio of normalized band intensity (G2 phase) of IP Hook2 to DIC in A (*n* = 4). **(C)** Ratio of normalized band intensity (G2 phase) of IP p150^glued^ to DIC in A (*n* = 4). **(D)** Lysates from HEK293T cells synchronized in prometaphase, metaphase/anaphase, and telophase and transfected with indicated siRNAs were IP with control IgG or antibodies against DIC or p150^glued^. The precipitates were IB with indicated antibodies. Arrows mark the lanes transfected with Hook2 siRNA. The bar graphs (on the right) represent normalized band intensity (control siRNA lane of respective cell cycle stage) of IP DIC to p150^glued^ and vice versa (*n* = 3). **(E)** Lysates from HEK293T cells synchronized in prometaphase and treated with control or Hook2 siRNA and transfected with EV or siRNA-resistant construct of Hook2 (WT/dynein binding-defective mutant) were tested for dynein–dynactin interaction as in D. Arrows mark the lanes transfected with Hook2 siRNA and siRNA-resistant Hook2 (WT). **(F)** Ratio of normalized band intensity (control siRNA) of IP DIC to p150^glued^ and vice versa in E (*n* = 2). Data represent mean ± SD (ns, not significant; ***, P < 0.001; Student’s *t* test).

Previous studies have shown that dynein regulates multiple stages of the cell cycle, including centrosome anchoring to the NE, chromosome alignment, spindle pole focusing, spindle positioning, and spindle assembly checkpoint inactivation (Howell et al., 2001; Salina et al., 2002; Goshima et al., 2005; Varma et al., 2008; Raaijmakers et al., 2012, 2013). To analyze the association of the Hook paralogs with dynein–dynactin during the cell cycle, we immunoprecipitated DIC and p150^glued^ from lysates of cells synchronized to different stages of the cell cycle. To this end, we synchronized cells using double-thymidine block and harvested them in different stages of cell cycle, namely, G2 phase (8 h after release), prometaphase (arrested by 100 µM nocodazole treatment 4 h after release), and late metaphase/early anaphase (10.5 h after release). While the p150^glued^ association with DIC remained unaltered during the cell cycle stages analyzed in this experiment (Fig. 2, A and C), Hook2 binding to dynein was significantly increased upon prometaphase onset (Fig. 2, A and B). In contrast, the interaction of Hook3 with dynein was detected during the G2 phase, but no detectable association was observed during prometaphase (Fig. 2 A). We were unable to detect Hook1 association with dynein and dynactin under endogenous conditions (Fig. 2 A). Interestingly, we noted that levels of both Hook1 and Hook3 were strikingly reduced at 10.5–11 h after release from double-thymidine block, whereas no significant differences in Hook2 levels were observed (Fig. 2 A and Fig. S2, A and C). The decrease in Hook1 and Hook3 levels coincided with the reduction of cyclin-B1 levels, which is a known substrate for anaphase promoting complex (APC)–mediated ubiquitin-proteasomal degradation and is degraded before the onset of anaphase (Chang et al., 2003). Hook1 and Hook3 protein expression were restored to detectable levels 13–15 h after release from thymidine block, a time point that corresponds to the early G1 stage (Fig. S2, A and C). As expected, restoration of Hook1 and Hook3 protein levels along with cyclin B1 required new protein synthesis, as the protein levels were not restored in cycloheximide-treated cells (Fig. S2, B and C). Bioinformatics analysis for potential APC recognition motif in the hook paralogs revealed a D-box (RXXLXXXXN) motif in Hook1 and Hook3, which is highly similar/identical to the D-box motif in Ninein-like protein-1, a known APC substrate (Fig. S2 D; Wang and Zhan, 2007). Whether this putative D-box motif in Hook1 and Hook3 mediates their degradation in an APC-dependent manner needs to be investigated in future work.

We next analyzed whether Hook2 influences dynein–dynactin association in a specific stage of cell cycle. Indeed, depletion of Hook2, but not Hook1 or Hook3, abrogated dynein–dynactin complex formation specifically during prometaphase and late metaphase/early anaphase stages of cell cycle (Fig. 2 D). Rescue of dynein–dynactin interaction in prometaphase cells by siRNA-resistant Hook2 WT, but not the dynein binding-defective mutants, confirmed that Hook2 is a crucial linker required for the dynein–dynactin association during mitosis (Fig. 2, E and F). We noted that both Hook2 and Hook3 were required for stable dynein–dynactin complex during the late cytokinesis/early G1 phase of the cell cycle; however, surprisingly, no significant change in the dynein–dynactin association was observed in Hook1 siRNA-treated cells (Fig. 2 D).

### Hook2 regulates anchoring of centrosomes to the NE

**Figure 3.**
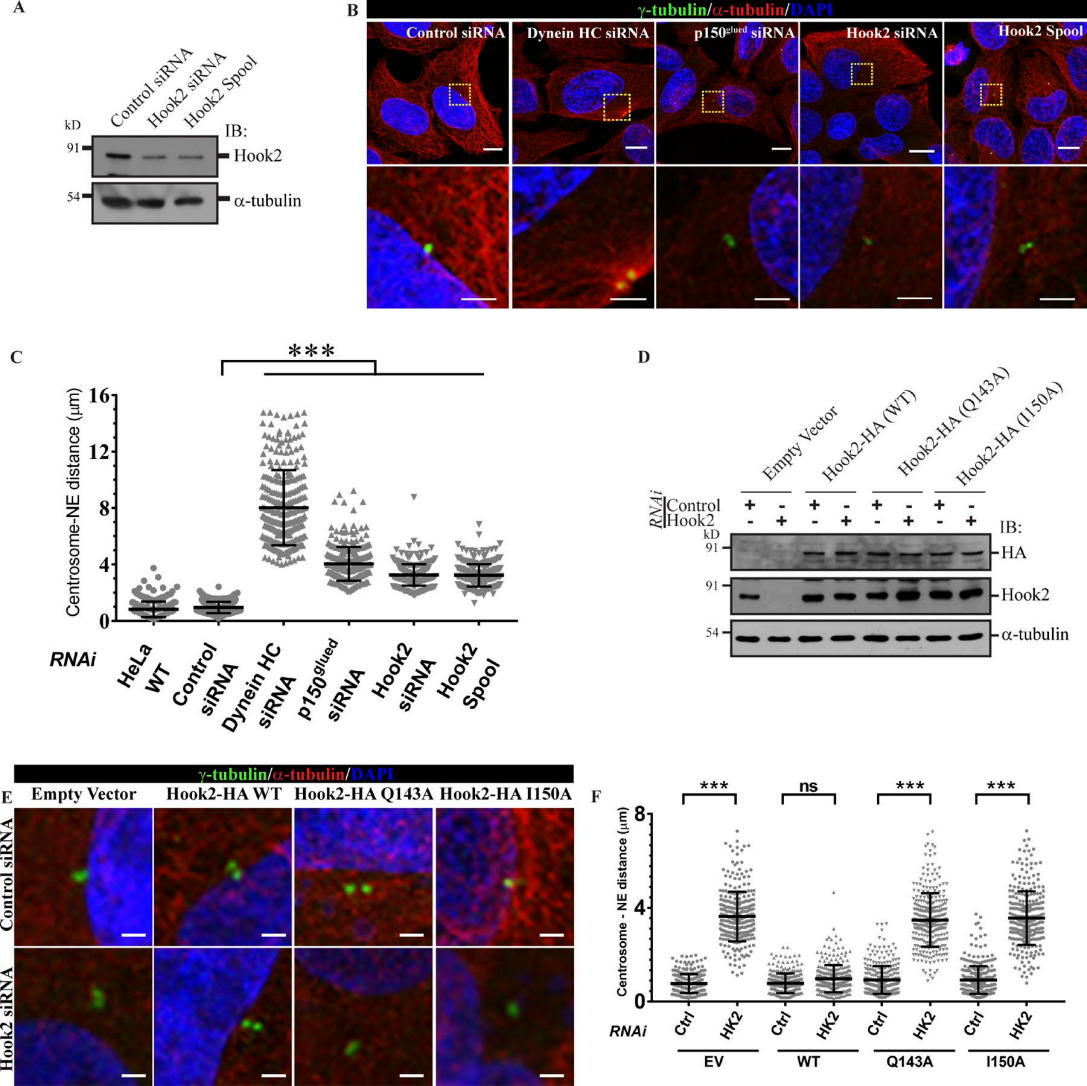
**Hook2 regulates anchoring of the centrosomes to the NE. (A)** HeLa cell lysates treated with indicated siRNA for 36 h were IB for Hook2 for assessing the knockdown efficiency, and α-tubulin was used as the loading control. **(B)** Representative images showing centrosome detachment from the nucleus of HeLa cells upon depletion of dynein, dynactin, and Hook2. Centrosomes are stained with γ-tubulin, MTs with α-tubulin, and nucleus with DAPI. Bars, upper images, 10 µm; lower zoomed insets, 2 µm. **(C)** Quantification of centrosome–NE distance in HeLa cells 36 h after siRNA transfections (*n* = 3; 150 centrosomes/experiment). **(D)** Western blot analysis with anti-HA and anti-Hook2 antibodies to confirm Hook2 knockdown and expression of siRNA-resistant constructs of Hook2 (WT and dynein binding-defective mutants) in control and Hook2 siRNA-treated HeLa cells. α-Gubulin was used as a loading control. **(E)** Representative images of rescue in centrosome attachment upon expression of siRNA-resistant Hook2 (WT) in Hook2-depleted HeLa cells but not with dynein-defective mutants. Bars, 2 µm. **(F)** Quantification of rescue in centrosome–NE distance as described in E (*n* = 3; 100 centrosomes/experiment). Data represent mean ± SD (ns, not significant; ***, P < 0.001; Student’s *t* test).

As Hook2 was required for dynein–dynactin association at the onset of mitosis, we next analyzed whether Hook2 regulates known functions of dynein during cell division. During late G2 and prophase stage of the cell cycle, dynein localizes to the NE in a dynactin-dependent manner and regulates centrosome anchoring to the NE and NE breakdown (Salina et al., 2002; Splinter et al., 2010; Raaijmakers et al., 2012, 2013). Indeed, whereas the distance between centrosome and NE was 0.83 ± 0.56 µm and 0.96 ± 0.39 µm in WT and control siRNA-treated HeLa cells, respectively, it was dramatically increased to 8.01 ± 2.63 µm and 4.05 ± 1.05 µm in dynein- and dynactin-depleted cells, respectively (Fig. 3 B and quantified in Fig. 3 C). To determine whether Hook2 also regulates centrosome attachment to the NE, we measured the distance between centrosome and NE in cells treated with either single siRNA oligo or pool of four oligos (SMARTpool [spool]) targeting Hook2 in HeLa cells. The efficiency of knockdown was ∼70% as confirmed by Western blotting (Fig. 3 A). Indeed, centrosome to NE distance was increased to 3.27 ± 0.77 µm and 3.24 ± 0.80 µm in cells treated with Hook2 siRNA and Hook2 spool, respectively (Fig. 3 B and quantified in Fig. 3 C). To evaluate whether Hook2 binding to dynein is required for centrosome–nucleus attachment, we expressed WT or Hook2 Q143A or I150A mutants in control and Hook2 siRNA-treated cells. The expression of all the constructs under experimental conditions was similar, as confirmed by Western blotting (Fig. 3 D). As shown in Fig. 3 E and quantified in Fig. 3 F, centrosome–nucleus attachment defects were rescued in Hook2 siRNA-treated cells transfected with WT Hook2 (vector: 3.63 ± 1.03 µm, Hook2 WT: 0.98 ± 0.55 µm) but not with Hook2 Q143A or I150A mutants (3.48 ± 1.44 µm and 3.56 ± 1.14 µm, respectively), suggesting that Hook2 binding to dynein–dynactin is required for mediating proper centrosome to NE attachment.

**Figure 4.**
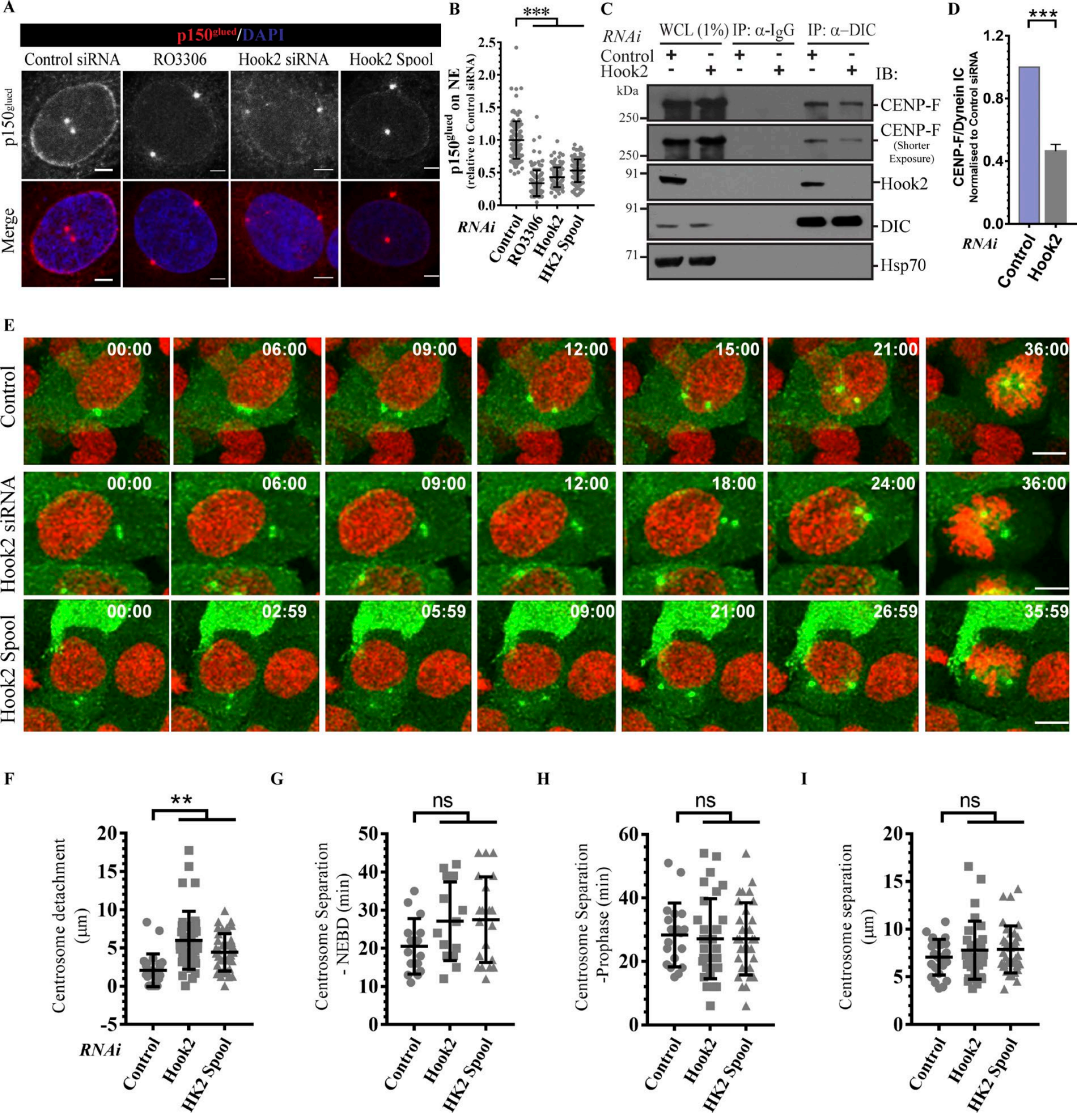
**Hook2 is required for p150^glued^ localization to the NE during the late G2 phase by regulating CENP-F–dynein interaction. (A)** Representative images showing loss of NE staining of p150^glued^ upon Hook2 depletion. Nucleus was visualized by DAPI. Bars, 2 µm. **(B)** Quantification of the intensity of NE staining of dynactin in A (*n* = 3; 40 cells/experiment). **(C)** Lysates from HEK293T cells treated with indicated siRNA and synchronized to late G2 phase were incubated with IP with control IgG or anti-DIC antibody. The precipitates were IB with indicated antibodies. Hsp70 was used as a loading control. **(D)** Ratio of normalized band intensity (control siRNA) of IP CENP-F to DIC in C (*n* = 3). **(E)** Maximum intensity projections of z-stacks of live-cell time-lapse imaging of HeLa cells stably expressing EB1-GFP and H2B-mCherry and transfected with indicated siRNA. Cells were imaged every 3 m to monitor centrosome detachment and separation before mitotic entry. Bars, 10 µm. **(F–I)** Quantifications of centrosome detachment from the nucleus (F), the duration between the start of centrosome separation to NEBD (G) or prophase end (H), and the distance between centrosomes at prophase end (I) as measured from live-cell imaging experiments shown in E and analyzed from 30 cells. Data represent mean ± SD (ns, not significant; **, P < 0.01; ***, P < 0.001; Student’s *t* test).

We next investigated whether Hook2 regulates dynein–dynactin localization at the NE, which in turn mediates anchoring of centrosomes to the NE. To this end, we synchronized cells using double-thymidine block and fixed them in the late G2 phase of the cell cycle when dynein–dynactin localization at the NE is readily observed (Salina et al., 2002). As illustrated in Fig. 4 A and quantified in Fig. 4 B, NE localization of the dynactin subunit p150^Glued^ was significantly reduced upon Hook2 depletion. We used the CDK1 inhibitor RO-3306 (9 µM) as a positive control in these experiments, as CDK1 has been previously shown to regulate dynein–dynactin NE localization (Baffet et al., 2015). Since dynactin is required for dynein localization at the NE (Raaijmakers et al., 2013), our observations also imply that dynein localization at the NE should be impaired. We could not directly visualize endogenous dynein localization at the NE due to poor staining with anti-DIC antibodies (data not shown). Although Hook2 localization at the centrosome and Golgi was obvious with an anti-Hook2 antibody (Fig. S2, E and F), we did not observe Hook2 localization at the NE (data not shown). This suggests that unlike its *Caenorhabditis elegans* orthologue Zyg12 that anchors at the NE, human Hook2 does not directly recruit dynein–dynactin to the NE (Minn et al., 2009). A previous study has shown that Hook2 via its first coiled-coil region interacts with the N-terminal domain of the large cell cycle–regulated protein, CENP-F (also known as mitosin; Moynihan et al., 2009). CENP-F has been previously shown to localize at the NE and mediate dynein–dynactin localization at the NE via binding to the nuclear pore complex protein Nup133 and dynein binding partners NudE and/or NudEL (Nde1/Ndel1; Bolhy et al., 2011). Since Hook2 binds dynein–dynactin and CENP-F through distinct domains, we investigated whether Hook2 promotes CENP-F–dynein interaction. We could detect a weak dynein–CENP-F complex by coIP using anti-DIC antibody (Fig. 4 C). Upon Hook2 depletion, we found consistently reduced interaction of CENP-F with dynein, indicating that at least one of the modes by which Hook2 mediates dynein–dynactin localization at the NE is by regulating dynein–CENP-F interaction (Fig. 4 C and quantified in Fig. 4 D).

To determine the consequences of whether centrosome detachment from the NE impacts mitotic progression, we analyzed centrosome separation kinetics during prophase in control and Hook2-depleted cells. To this end, we performed confocal time-lapse microscopy of control and Hook2-depleted HeLa cells stably expressing H2B-mCherry and EB1-GFP (Fig. 4 E and Video 1). We found that EB1-GFP accumulates at the nucleating centrosome and, similar to the previously described EB3-GFP (Bolhy et al., 2011), was a suitable marker for determining centrosome dynamics during mitosis. Although centrosome detachment from the NE was readily observed in time-lapse imaging of Hook2-depleted cells (Fig. 4 E and Video 1, and quantified in Fig. 4 F), we did not find any significant differences between control and Hook2-depleted cells in the time taken from centrosome splitting to NEBD (Fig. 4 G) or until the end of prophase (Fig. 4 H), as visualized by penetration of spindle MTs into the chromosome mass following NEBD. The final mean distance between the two centrosomes at the end of prophase was also similar in control and Hook2-depleted cells (7.2 ± 1.7 µm in control, 8.3 ± 3.05 µm in Hook2 siRNA, and 8 ± 2.5 µm in Hook2 spool-treated cells [Fig. 4 I]). Thus, the NE disengagement of centrosomes, just before NEBD, does not appear to affect proper and timely bipolar spindle formation in Hook2-depleted cells. Our findings are in agreement with a previous report showing that bipolar spindle formation, chromosome congression, and segregation proceeded normally in cells lacking NE localization of CENP-F and dynactin (Bolhy et al., 2011).

### Hook2 depletion results in chromosome congression and spindle positioning defects

**Figure 5.**
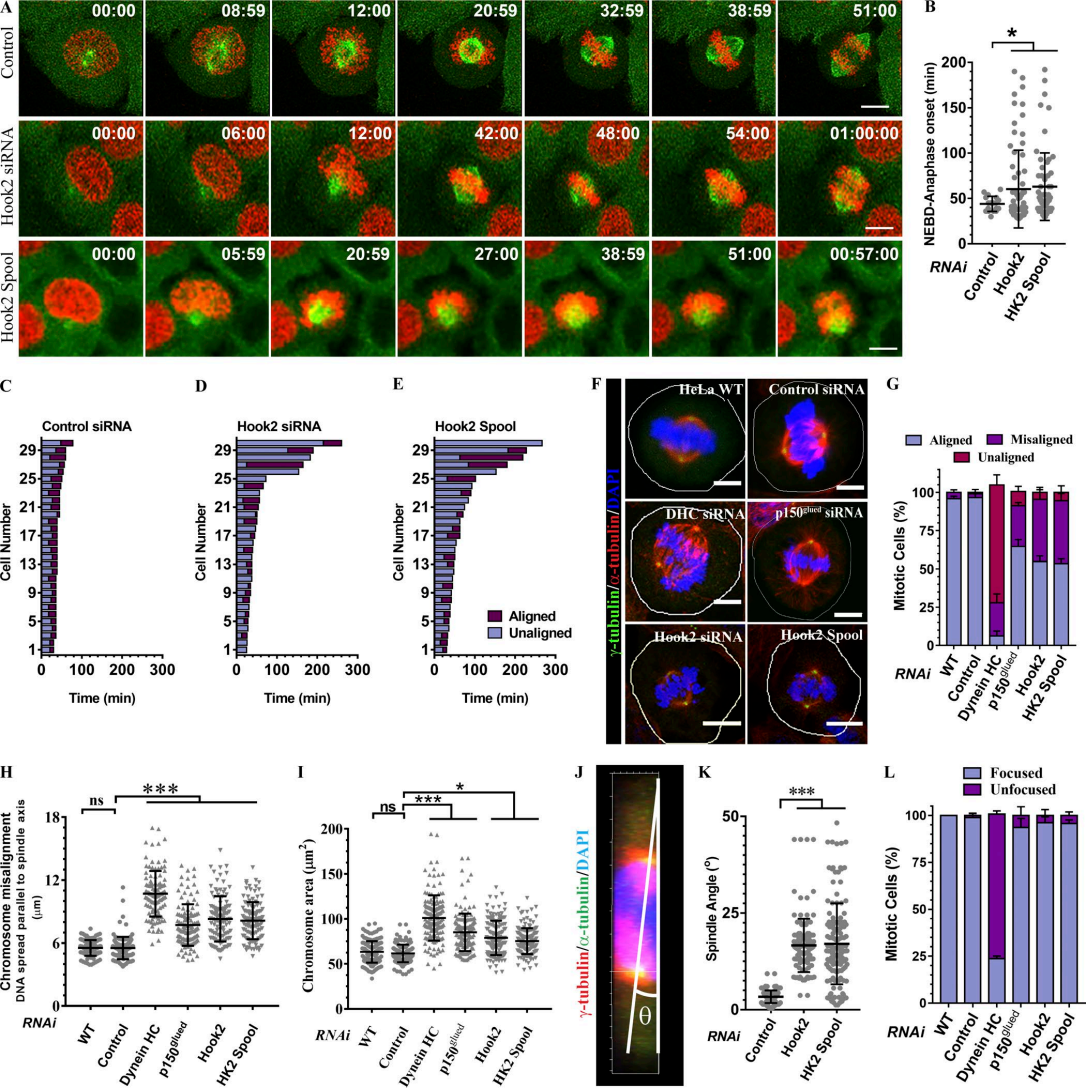
**Hook2 depletion results in mitotic progression defects. (A)** Maximum intensity projections of z-stacks of live-cell time-lapse imaging from HeLa cells stably expressing GFP–α-tubulin and H2B-mCherry and transfected with indicated siRNA. Cells were imaged every 3 m to monitor mitotic progression in each case. Bars, 10 µm. **(B)** Quantification of time taken from the NEBD to anaphase onset in cells transfected with control or Hook2 siRNA measured (*n* = 2; 40 mitotic events/experiment). **(C–E)** Quantification of mitotic timing and chromosome alignment of live-cell imaging experiment shown in A. Asynchronous cells were imaged for the duration of 12 h, and images were acquired every 3 m. Bars in the graph represent total time spent in mitosis for individual cells from a single experiment. Gray bars indicate time spent with unaligned chromosomes, and violet bars indicate time spent with full chromosome alignment. The starting point is NEBD, and the end of the bar represents either anaphase onset or death in mitosis (*n* = 2; 15 cells/experiment). **(F)** Representative images of mitotic HeLa cells treated with indicated siRNA. Centrosomes were stained with γ-tubulin and MTs with α-tubulin, and chromosomes were visualized with DAPI. Bars, 5 µm. **(G)** Quantification of chromosome misalignment from cells described in F (*n* = 3; 100 mitotic cells/experiment). **(H)** The extent of chromosome misalignment shown as a dot plot. To calculate the extent of chromosome misalignment, the DNA spread parallel to spindle pole axis was measured using ImageJ software (*n* = 3; 100 mitotic cells/experiment). **(I)** The extent of chromosome congression shown as a dot plot. To calculate the extent of chromosome congression, the area of DNA spread inside each mitotic cell was measured using ImageJ software (*n* = 3; 100 mitotic cells/experiment). **(J)** Schematic showing the calculation of spindle positioning defect measured as a function of an angle between spindle pole axis and substratum. **(K)** Quantification of spindle positioning defect upon Hook2 depletion in HeLa cells (*n* = 3; 35 metaphase cells/experiment). **(L)** Quantification of spindle pole focusing in HeLa cells treated with indicated siRNAs (*n* = 3; 100 metaphase cells/experiment). Data represent mean ± SD (ns, not significant; *, P < 0.1; ***, P < 0.001; Student’s *t* test).

We next analyzed mitotic progression in Hook2-depleted cells beyond prometaphase. To this end, we performed time-lapse video imaging of control and Hook2-depleted HeLa cells stably expressing H2B-mCherry and GFP–α-tubulin (Fig. 5 A and Videos 2, 3, and 4). Live-cell imaging revealed significant chromosome congression defects in ∼20% of Hook2-depleted cells (Fig. 5, C–E), with a concomitant increase in the time duration from NEBD to anaphase onset (Fig. 5 B). We quantified the chromosome congression defects from ∼100 mitotic HeLa cells that were pretreated with control siRNA or siRNA against Hook2, dynein, and dynactin and fixed before imaging (representative images shown in Fig. 5 F). While in WT and control siRNA-treated cells, ∼5% of the cells showed one or more chromosomes unaligned at the metaphase plate, upon Hook2 depletion, ∼45% of cells had the similar defect in chromosome alignment, with ∼6% of cells showing completely unaligned chromosomes (Fig. 5 G). Further, DNA spread over an average distance of 8.31 ± 2.15 µm and 8.14 ± 1.77 µm parallel to the spindle pole axis in cells depleted of Hook2 with siRNA or spool, respectively (Fig. 5 H). This was significantly more than WT and control siRNA-treated cells, where the average distance of DNA spread was 5.54 ± 0.75 µm and 5.53 ± 1.07 µm, respectively (Fig. 5 H). Accordingly, the area of DNA spread was increased by ∼22–28% in Hook2-depleted cells as compared with the control (Fig. 5 I). We also noted that Hook2-depleted cells had severe spindle orientation defects, as evident by increased spindle angle (angle between spindle pole axis [straight line spanning the centrosomes] and substratum) compared with control cells (Fig. 5, J and K).

Expectedly, in dynein-depleted cells, chromosome congression defects were significantly enhanced, with ∼75% of cells showing gross defects in chromosome alignment (Fig. 5, F and G). DNA in dynein-depleted metaphase cells was spread over a distance of 10.70 ± 2.17 µm (Fig. 5 H). Dynactin depletion had a less pronounced effect on chromosome alignment than depletion of dynein, with defects observed in ∼28% of cells (Fig. 5, F and G). The DNA was spread over a distance of 7.72 ± 1.98 µm in dynactin-depleted cells (Fig. 5 H). The area of DNA spread was also accordingly increased in case of dynein and dynactin depletion by ∼64% and ∼39%, respectively (Fig. 5 I). We also scored metaphase cells treated with different siRNAs for focused and unfocused spindles, an aspect of spindle organization that depends upon dynein function (Fig. 5 L). Spindle pole focusing defects were not observed in Hook2- or dynactin-depleted cells, whereas, as noted in the earlier studies (Goshima et al., 2005; Raaijmakers et al., 2013), dynein depletion severely abrogated formation of focused bipolar spindles (Fig. 5 L).

### Hook2 regulates MT nucleation

To understand the mechanism of chromosome mis-congression and spindle mis-orientation upon Hook2 depletion, we first analyzed whether Hook2 regulates dynein localization to kinetochore (KT) and the cell cortex. Dynein localization at the KTs is crucial for chromosome movement toward spindle poles and for the establishment of stable KT-MT attachments that regulates chromosome congression and segregation (Yang et al., 2007; Amin et al., 2018). KT-associated dynein also plays a crucial role in stripping spindle assembly checkpoint proteins and promoting anaphase onset (Howell et al., 2001; Wojcik et al., 2001). During metaphase, dynein localizes to the cell cortex and exerts pulling forces on the plus ends of astral MTs for correct spindle positioning (Kiyomitsu and Cheeseman, 2012; Kotak and Gönczy, 2013). As shown in Fig. S3, endogenous DIC levels at the KT (Fig. S3, A and B) and cortex (Fig. S3, C and D) were similar in control and Hook2-depleted cells, while no detectable DIC staining at KT and cortex was observed upon p150^glued^ depletion, as previously reported (Kiyomitsu and Cheeseman, 2012; Raaijmakers et al., 2013; Fig. S3, A–D). This is not surprising as dynein localization at the cortex and KTs is regulated by other dynein–dynactin binding partners, including the cortically anchored NuMA-LGN-Gαi complex and the KT-associated dynein–dynactin adaptor, Spindly, respectively (Griffis et al., 2007; Kiyomitsu and Cheeseman, 2012; Kotak and Gönczy, 2013). Indeed, unlike Spindly, which localizes to the KT, we did not observe Hook2 localization at the KT (Fig. S3, E and F).

**Figure 6.**
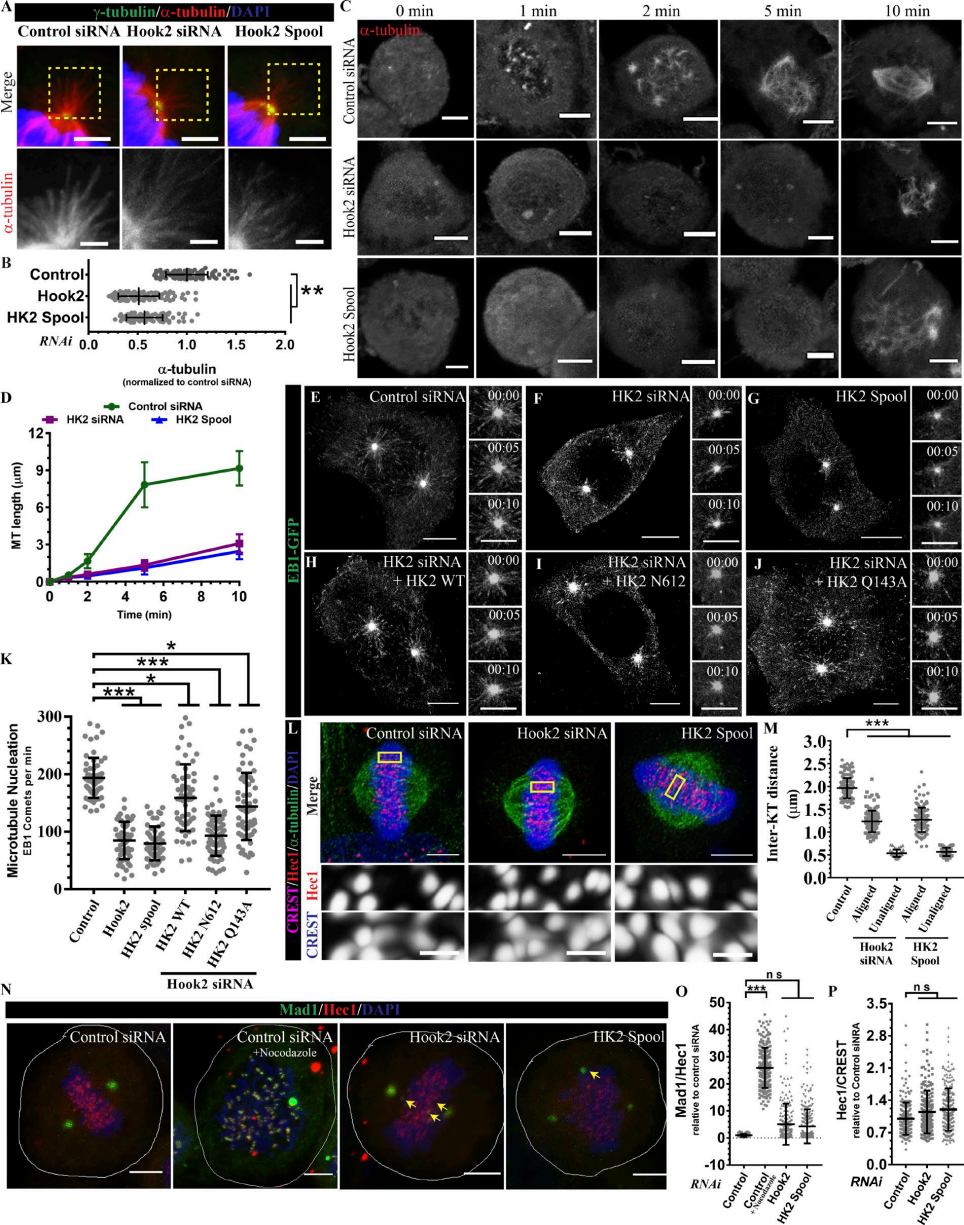
**Hook2 regulate MT nucleation at the centrosome. (A)** Representative images of metaphase HeLa cells transfected with indicated siRNA zoomed to visualize astral MTs. Bars, 2 µm. **(B)** Quantification of the intensity of astral MTs in A (*n* = 3; 40 metaphase cells/experiment). **(C)** Representative image of MT regrowth upon cold depolymerization in mitotic HeLa cells transfected with indicated siRNA. Bar, 5 µm. **(D)** Quantification of MT length as shown in C (*n* = 3; 30 cells/experiment). **(E–J)** Maximum intensity projections of z-stacks of live-cell time-lapse imaging of early prophase HeLa cells stably expressing EB1-GFP and treated with indicated siRNA and transfected with respective siRNA-resistant Hook2 (WT/mutants) constructs as indicated. Images were acquired every 5 s for a total duration of 5 m. Bars, image, 10 µm; zoomed insets, 2 µm. **(K)** Quantification of the rate of MT nucleation from centrosomes as determined from the time-lapse videos shown in E through J from 40 cells (two centrosomes/cell). **(L)** Representative maximum intensity projection of airy-scan images of metaphase-arrested HeLa cells treated with indicated siRNA. MTs were stained with α-tubulin, KTs were stained with CREST and Hec1 antibodies, and chromatin was visualized with DAPI. Bars, upper images, 5 µm; lower insets, 2 µm. **(M)** Quantification of inter-KT distance from 10 metaphase cells (30 KT pairs/cell, representative image shown in L). **(N)** Representative images of metaphase-arrested HeLa cells treated with indicated siRNA. KTs were stained with Hec1 antibody, and silencing of the mitotic checkpoint was confirmed by the Mad1 antibody. Prometaphase-arrested (100 µM nocodazole) control siRNA-treated cells were taken as a positive control in the experiment. **(O)** Quantification of silencing of mitotic checkpoint upon Hook2 siRNA from cells depicted in N from 20 cells (10–15 KTs/cell). **(P)** Quantification of Hec1 levels at the KTs in HeLa cells from 10 metaphase cells (30 KTs/cell, representative image shown in L). Data represent mean ± SD (ns, not significant; *, P < 0.1; **, P < 0.01; ***, P < 0.001; ****, P < 0.0001; Student’s *t* test).

We noted that Hook2-depleted cells had approximately twofold reduced astral MT staining that was quantified by measuring α-tubulin intensity proximal to the cell cortex (Fig. 6, A and B). MT nucleating factors at the centrosome, including γ-tubulin ring complexes (γTuRCs) and their regulators, mediate astral MT nucleation (Kollman et al., 2011). Previous work has suggested that Hook2 is also required for MT nucleation, as overexpression of Hook2 truncation mutants (lacking either the Hook domain or C-terminal tail) impairs MT regrowth after depolymerization with nocodazole (Szebenyi et al., 2007). Consistent with the prior findings, we noted that MT regrowth at different time points after cold depolymerization was significantly reduced upon Hook2 depletion (Fig. 6, C and D; MT length ∼8 µm in control versus 2 µm in Hook2-depleted cells 5 min after incubation at 37°C). We also noted that while control cells showed multiple MT asters at 5 min after incubation, there were no MT asters observed in Hook2-depleted cells at these time points (Fig. 6 C).

To examine the dynamics of MT nucleation, we measured the number of EB1-GFP comets emerging from the centrosomes per unit time (rate) in control and Hook2-depleted cells specifically during the prophase-prometaphase transition. EB1 marks the plus ends of growing MTs and labels newly nucleated MTs, accordingly, measuring the rate of EB1-GFP comets is a well-established approach to measure MT growth (Piehl et al., 2004; Salaycik et al., 2005). As illustrated in snapshots of Video 5 (Fig. 6, E–J) and quantified in Fig. 6 K, we found an ∼50% decrease in the rate of EB1 comet emergence in Hook2-depleted cells as compared with the control cells. The siRNA-resistant Hook2 (WT), but not a truncation mutant (N612) that does not localize at the centrosome (Fig. S3 G), was able to partially rescue MT nucleation defects (Video 6; Fig. 6, H and I; and quantified in Fig. 6 K). Notably, dynein binding-defective mutant of Hook2 (Q143A) that continues to localize at the centrosome (Fig. S3 G) rescued MT nucleation defect similar to Hook2 WT, suggesting that centrosomal localization of Hook2, but not its dynein binding function, is required for mediating normal rates of MT nucleation (Video 6 and Fig. 6 J and quantified in Fig. 6 K).

Reduced astral MT nucleation can lead to the defective anchoring of at least one of the spindle poles (also consistent with the spindle mis-positioning phenotype observed upon Hook2 depletion; Fig. 5, J and K), which would lead to a failure to establish proper KT-MT tension due to the loosely anchored pole(s). Indeed, the average inter-KT distance was 1.23 ± 0.23 µm and 1.26 ± 0.27 µm in Hook2 single oligo and spool-treated cells, respectively, as compared with 1.96 ± 0.22 µm in control cells (Fig. 6 L and quantified in Fig. 6 M). The reduced inter-KT distance indicates that KTs are under partial tension upon Hook2 depletion. Further, we quantified the levels of Mad1, a MT attachment-sensing checkpoint protein at the KTs. Mad1 was retained on few aligned KTs upon Hook2 depletion, also suggesting a defect in KT-MT attachment (Fig. 6 N and quantified in Fig. 6 O). Our results show that Hook2 does not localize to KTs (Fig. S3, E and F); moreover, the levels of KT-associated dynein (Fig. S3 B) and of Hec1, a subunit of the Ndc80 complex that mediates end-on KT-MT attachment, were similar in control and Hook2-depleted cells (Fig. 6 L and quantified in Fig. 6 P). Therefore, it is likely that suboptimal KT-MT attachments observed upon Hook2 depletion are primarily because of reduced MT nucleation rather than due to defective attachment.

We next investigated how Hook2 regulates MT nucleation from the centrosome. During prometaphase, centrosomal localization of γ-tubulin (MT nucleating factor at the centrosomes) and pericentrin (which anchors the nucleating factors to the centrosome) was not affected upon Hook2 depletion, although treatment with Polo-like kinase 1 inhibitor (BI-2536), as expected, significantly reduced γ-tubulin and pericentrin centrosomal levels (Barr et al., 2004; Lee and Rhee, 2011; Fig. S3 H; and quantified in Fig. S3, I and J). Further, the dynein–dynactin motor complex that transports the nucleating factors to centrosome during late G2 phase continued to localize at the centrosome upon Hook2 depletion (Young et al., 2000; Fig. S3, K and L; and quantified in Fig. S3, M and N). Accordingly, centrosomal localization of γ-tubulin and pericentrin during the late G2 phase was not altered upon Hook2 depletion (Fig. S3, K and L; and quantified in Fig. S3, O and P). The precise mechanisms underlying Hook2-mediated MT nucleation from the mitotic centrosomes remain an open question that will require further detailed exploration.

### Hook2 depletion prevents complete furrow ingression resulting in cytokinesis failure

**Figure 7.**
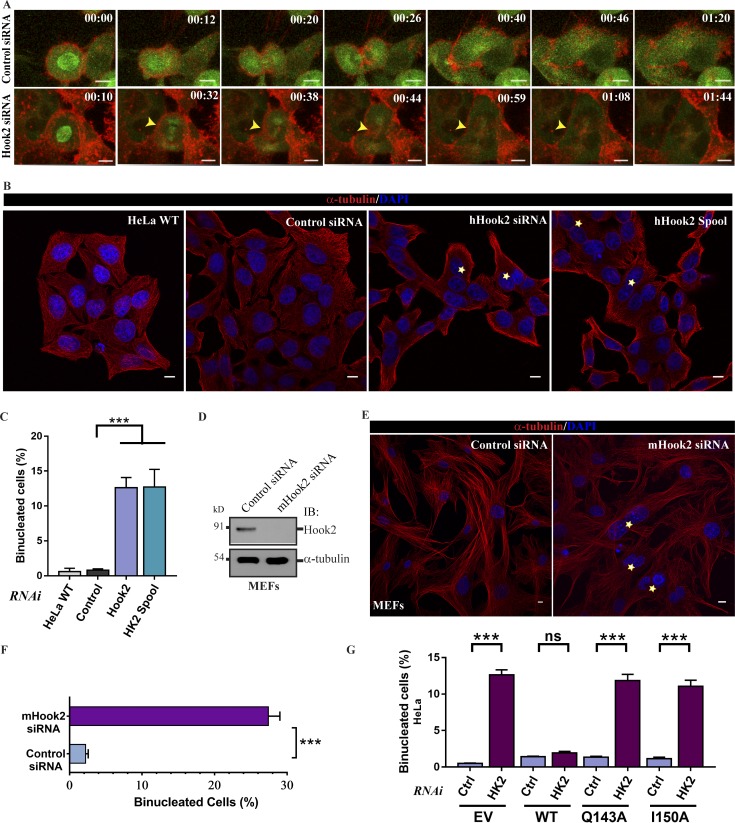
**Hook2 depletion causes cytokinesis failure. (A)** Maximum intensity projections of z-stacks of live-cell imaging of HeLa cells stably expressing GFP–α-tubulin and mCherry-UtrCH and treated with control or Hook2 siRNA. Z-stack time-lapse images were acquired every 3 m for a total duration of 3 h. Bars, 10 µm. **(B)** Representative images of HeLa cells treated with indicated siRNAs. The yellow asterisks in the images indicate binucleated cells in each case. Bars, 10 µm. **(C)** Quantification of binucleated HeLa cells as described in B (*n* = 5; 300 cells/experiment). **(D)** Western blot analysis confirming depletion of Hook2 in primary MEFs, and α-tubulin was used as a loading control. **(E)** Representative images of primary MEFs treated with control or mHook2 siRNA. The yellow asterisks in the images indicate binucleated cells in each case. Bars, 10 µm. **(F)** Quantification of binucleated primary MEFs as described in E (*n* = 5; 300 cells/experiment). **(G)** Quantification of the percentage of binucleated cells in siRNA-resistant Hook2 (WT/Q143A/I150A) transfected HeLa cells treated with control or Hook2 siRNA (*n* = 3; 200 cells/experiment). Data represent mean ± SD (ns, not significant; ***, P < 0.001; Student’s *t* test).

Time-lapse video imaging of Hook2-depleted cells showed that the majority of cells (∼60%) with or without mitotic delay initiated cleavage furrow formation that later regressed, resulting in the formation of binucleated cells (Fig. S4 A, and quantified in Fig. S4 B and Videos 7, 8, and 9). The defect in the completion of furrow ingression was confirmed by time-lapse imaging of cells expressing GFP–α-tubulin and cortical actin marker, mCherry-UtrCH (calponin homology domain of Utrophin), which does not alter actin dynamics (Burkel et al., 2007; Fig. 7 A and Video 10). We corroborated our findings in asynchronous HeLa cells and primary mouse embryonic fibroblasts (MEFs), where a significant increase in the fraction of binucleate cells was observed upon Hook2 depletion, as compared with the control (Fig. 7, B–F; and quantified in Fig. 7, C and F). While siRNA-resistant Hook2 (WT) rescued the binucleated phenotype, no rescue was observed with the dynein binding-defective mutants (Fig. 7 G), suggesting that Hook2 function as the dynein–dynactin linker is crucial for its role in furrow ingression.

### Hook2 localizes to central spindles and promotes recruitment of dynein and dynactin to central spindles

**Figure 8.**
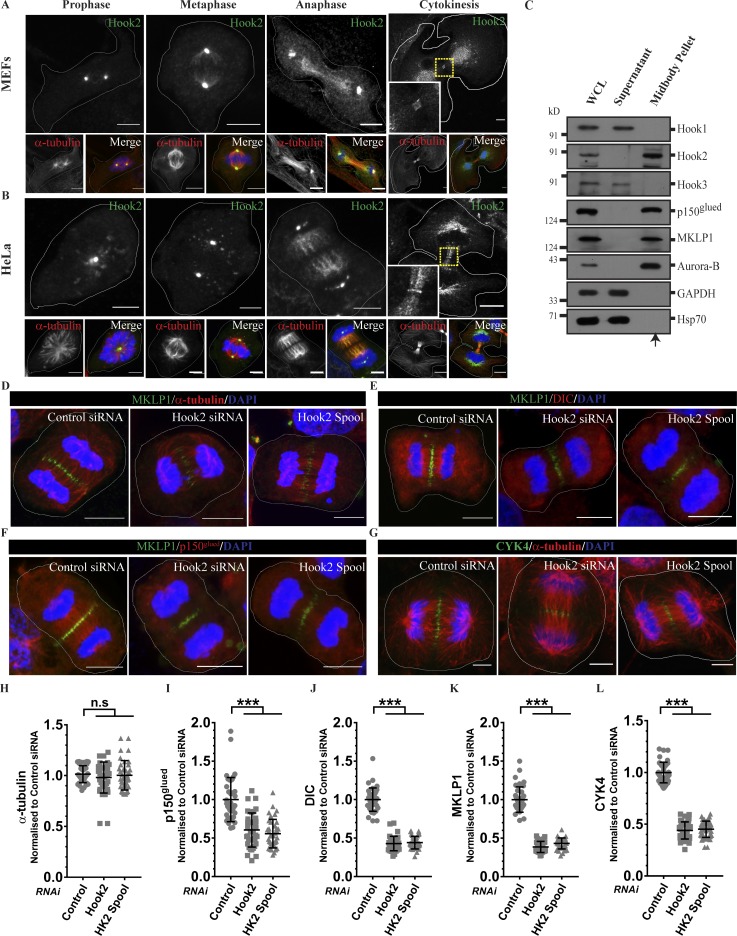
**Hook2 localizes to the central spindles and promotes recruitment of dynein and dynactin to the central spindles. (A and B)** Representative images of primary MEFs (A) and HeLa cells (B) in indicated stages of the cell cycle stained for endogenous Hook2, MTs with α-tubulin, and chromosomes with DAPI. Bars, 10 µm. **(C)** Western blot showing enrichment of Hook2 in midbody pellet from synchronized HeLa cells as compared with supernatant containing cytosol from the same cells. **(D–G)** Representative images of HeLa cells in anaphase treated with indicated siRNA. MTs were stained with α-tubulin to visualize spindles. Dynein, dynactin, and spindle midzone were visualized by antibodies against DIC, p150^glued^, CYK4 and MKLP1, respectively, and chromosomes were visualized by DAPI in each case. Bars, 10 µm. **(H–L)** Quantification of the relative levels (control siRNA) of α-tubulin, p150^glued^, DIC, MKLP1, and CYK4 at central spindles and spindle midzone, respectively (*n* = 3; 15 cells/experiment). Data represent mean ± SD (ns, not significant; ***, P < 0.001; Student’s *t* test).

To gain insights into the Hook2 function in regulating cytokinesis, we first evaluated Hook2 localization during anaphase and cytokinesis. Consistent with earlier work (Szebenyi et al., 2007), we observed Hook2 localization on centrosomes throughout the cell cycle (Fig. 8, A and B). Surprisingly, we also found Hook2 localization on the central spindles and midbody ring during late anaphase and cytokinesis in multiple cell lines, including MEFs (Fig. 8 A) and HeLa cells (Fig. 8 B). The central spindles and midbody ring staining were highly reduced in Hook2 siRNA-treated HeLa cells, confirming the presence of Hook2 on these structures (Fig. S4 C and quantified in Fig. S4 D). Interestingly, we noted that the midbody ring placement in the intercellular bridge became asymmetric upon Hook2 depletion (as evident in Fig. S4 C; quantification of α-tubulin staining is shown in Fig. S4 D). The significance of this observation is not clear and needs to be explored in future studies. Finally, we performed biochemical purification of midbodies that also confirmed the presence of Hook2 in the midbody pellet along with other well-characterized midbody proteins, such as mitotic kinesin-like protein 1 (MKLP1) and Aurora B kinase (Fig. 8 C). The purity of the midbody fraction was confirmed by probing for GAPDH and Hsp70, which are predominantly cytosolic proteins and were detected in the supernatant but not in the midbody fraction. Notably, of the three Hook paralogs, only Hook2 was present in the midbody fraction (Fig. 8 C).

Previous reports have shown that p150^glued^ localizes to the central spindles and regulates central spindle organization (Delcros et al., 2006; Reboutier et al., 2013). We did not observe changes in α-tubulin intensity on the central spindles upon Hook2 depletion; however, dynactin and dynein levels on the central spindles were significantly reduced in Hook2-depleted cells as compared with the control (Fig. 8, D–F; and quantification shown in Fig. 8, H–J). This was better visualized in cytokinetic cells (HeLa and MEFs), where dynactin levels on the MTs forming the cytokinetic bridge were significantly reduced upon Hook2 depletion (Fig. S4, E–H).

### Hook2 regulates dynactin-dependent targeting of centralspindlin complex to the midzone

Dynactin on the central spindles regulates localization of the centralspindlin complex subunit MKLP1/Kif23 (known as Pavarotti in *Drosophila melanogaster*) to the midzone as shown in *Drosophila* S2 cells (Delcros et al., 2006). MKLP1 is plus end–directed kinesin-6 protein that, along with Rho family GTPase-activating protein (CYK4), forms the centralspindlin complex, which activates RhoA to mediate cleavage furrow ingression (Adams et al., 1998; Hirose et al., 2001; Mishima et al., 2002). In agreement with these studies, we observed highly reduced intensity of MKLP1 staining at the midzone in p150^glued^-depleted HeLa cells that had escaped into anaphase, although the majority of cells were still arrested in metaphase (Fig. S4 I and quantified in Fig. S4 J). These findings suggest that dynactin targets MKLP1 to the midzone in human cells as well. Consistent with our observation that Hook2 regulates p150^glued^ levels on the central spindle, we noted that upon Hook2 depletion, the fluorescence intensity of both centralspindlin components, MKLP1 and CYK4, was reduced at the spindle midzone (Fig. 8, E–G; and quantification shown in Fig. 8, K and L). The loss of MKLP1 fluorescence from midzone was less dramatic in Hook2-depleted cells, as compared with p150^glued^ depletion (compare Fig. 8 K with Fig. S4 J), likely due to remaining levels of p150^glued^ on the central spindle upon Hook2 depletion (Fig. 8 I).

**Figure 9.**
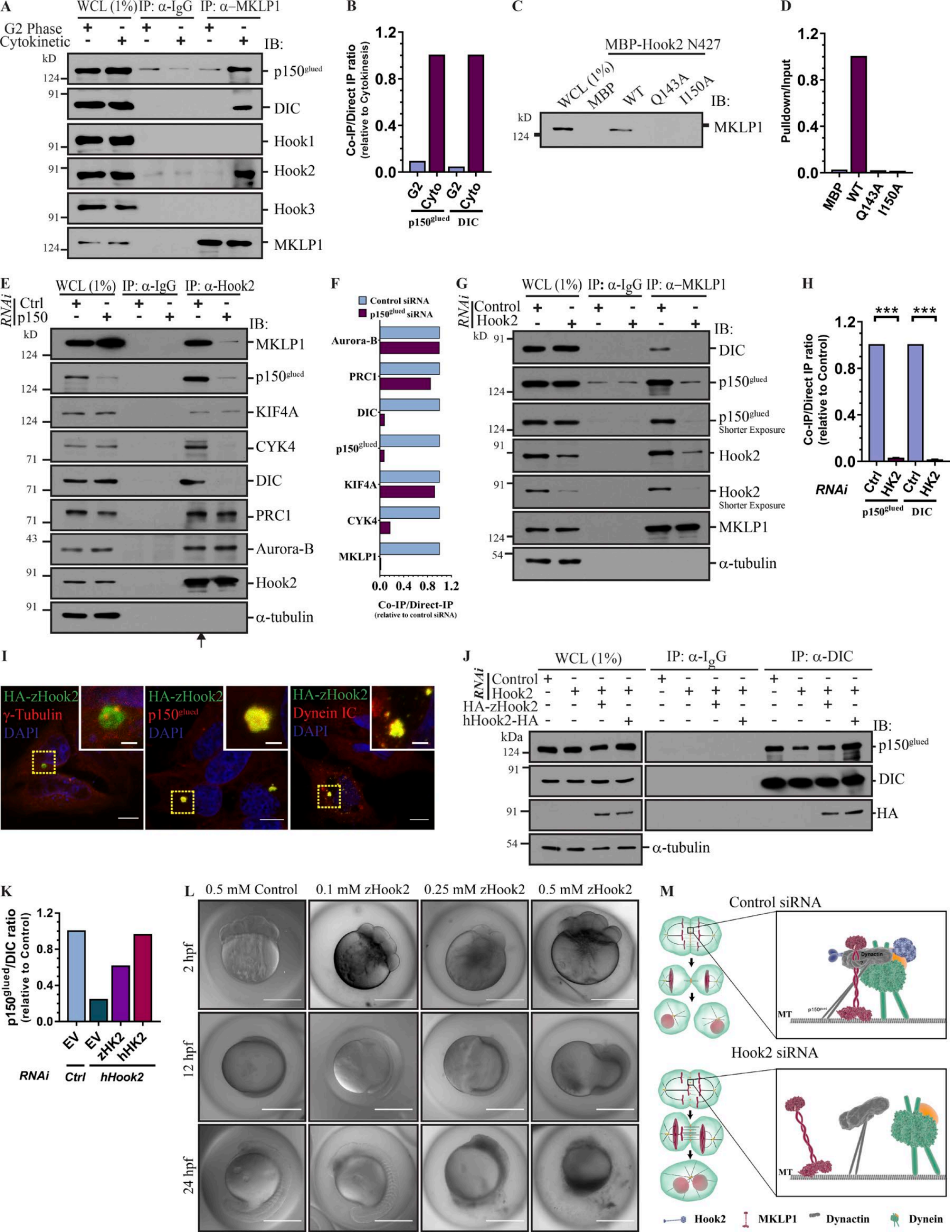
**Hook2 is required for p150^glued^ and MKLP1 interaction during cytokinesis. (A)** Lysates of HEK293T cells synchronized in either G2 phase or cytokinesis were IP with control IgG or anti-MKLP1 antibody and IB with indicated antibodies. **(B)** Ratio of normalized band intensity (cytokinesis lane) of IP p150^glued^ and DIC to MKLP1 in A (*n* = 2). **(C)** Whole-cell lysates of HEK293T cells harvested during the cytokinesis stage were incubated with either MBP or MBP-tagged Hook2 N427 (WT, Q143A, and I150A) and IB for MKLP1. **(D)** Ratio of band intensity of pulldown to input Hook2 (WT/mutants) signal in C (*n* = 2). **(E)** Lysates of HEK293T cells treated with control or p150^glued^ siRNA and harvested during cytokinesis stage were IP with control IgG or anti-Hook2 antibody and IB with the indicated antibodies. **(F)** Ratio of normalized band intensity (control siRNA) of coIP proteins (as indicated) to MKLP1 in E (*n* = 2). **(G)** Lysates of HEK293T cells transfected with indicated siRNA and harvested during cytokinesis stage were tested for dynactin association with MKLP1. **(H)** Ratio of normalized band intensity (control siRNA) of IP p150^glued^ and DIC to MKLP1 in G (*n* = 3). **(I)** Representative images of HeLa cells ectopically expressing HA-tagged zebrafish Hook2 (HA-zHook2) costained for centrosomes, dynein, and dynactin. Bars, 10 µm; insets, 2 µm. **(J)** Lysates from asynchronous HEK293T cells treated with indicated siRNA and transfected with indicated plasmids were IP with control IgG or anti-DIC antibody and IB for p150^glued^. **(K)** Ratio of normalized band intensity (control siRNA) of IP p150^glued^ to DIC in J (*n* = 2). **(L)** Representative differential interference contrast images of zebrafish embryos injected with either nontargeting control morpholino or indicated concentration of zHook2 morpholino immediately after fertilization and imaged at the indicated time. Each concentration of morpholino was injected in 100 fertilized embryos and monitored over time. Bars, 500 µm. Data represent mean ± SD (ns, not significant; ***, P < 0.001; Student’s *t* test). **(M)** Proposed model depicting the role of Hook2 in mediating formation of dynein–dynactin complex and targeting of MKLP1 to the spindle midzone.

To understand how Hook2 might enable MKLP1 localization at the midzone, we tested whether MKLP1 and dynactin are present in a complex and whether Hook2 is required for their stable association. We compared these protein–protein interactions in lysates from HEK293T cells enriched in G2 phase or cytokinesis stages of the cell cycle. Indeed, both Hook2 and dynactin were IP with MKLP1 only during cytokinesis, suggesting that these interactions likely occur upon recruitment of the proteins on the central spindles (Fig. 9 A and densitometric quantification shown in Fig. 9 B). Dynein subunit DIC was also coIP with MKLP1, although whether dynein has a functional role as part of this complex remains unclear (Fig. 9 A). Notably, we did not see Hook1 and Hook3 coIP with MKLP1, suggesting that MKLP1 specifically interacts with dynactin and Hook2 complex (Fig. 9 A). To corroborate our observations, we used an independent approach where lysates from HEK293T cells enriched in the cytokinesis phase were incubated with either MBP-tagged Hook2 N427 WT or the LIC binding-defective Hook2 point mutants. As shown in Fig. 9 C (densitometric quantification is shown in Fig. 9 D), pulldown of MKLP1 was observed with the WT (that interacts with p150^glued^) but not the LIC binding-defective mutants of Hook2. Hook2 interaction with MKLP1 was abrogated in p150^glued^-depleted cells, suggesting that Hook2 did not directly bind to MKLP1 (Fig. 9 E and quantified in Fig. 9 F). We also probed these eluates for other components of the central spindle and midbody, namely, CYK4 (subunit of centralspindlin complex), PRC1 (MT cross-linking protein that is associated with midbody MTs), KIF4 (chromokinesin and PRC1-binding partner), and Aurora B kinase (subunit of chromosome passenger complex; Fig. 9 E and quantified in Fig. 9 F). We found that Hook2 was associated with all of these components of the central spindle/midbody; however, only the centralspindlin complex (MKLP1 and CYK4) association was reduced upon p150^glued^ depletion (Fig. 9 E). Our repeated attempts at detecting a direct binding between MKLP1 and p150^glued^ were not successful (data not shown), which might be because other subunits of the dynactin complex are involved in direct binding to the centralspindlin complex.

We then analyzed whether Hook2-mediated dynein–dynactin targeting at the central spindle is prerequisite for dynactin and MKLP1 association during cytokinesis. Indeed, MKLP1 interaction with p150^glued^ was considerably reduced upon Hook2 depletion (Fig. 9 G and quantified in Fig. 9 H). We confirmed these interactions in reverse orientation as well where coIP of MKLP1 with p150^glued^ was observed in cytokinetic cells, only in the presence of Hook2 (Fig. S5 A and quantified in Fig. S5 B). Hook3 depletion did not affect MKLP1 interaction with dynactin, supporting our previous data that Hook2, but not other Hook paralogs, forms complexes with MKLP1 and dynactin (Fig. S5 C and quantified in Fig. S5 D). Taken together, our findings suggest that Hook2 stabilizes cleavage furrow ingression by promoting dynactin, and consequently, MKLP1 localization on the central spindle and spindle midzone, respectively (Fig. 9 M).

### Zebrafish Hook2 homologue rescues dynein–dynactin association and is essential for early development

To understand whether Hook2 function as a linker between dynein–dynactin is conserved across evolution, we analyzed the localization and dynein association of Hook2 homologue in zebrafish (zHook2) that is functionally uncharacterized hitherto. Zebrafish have two Hook paralogs, of which one is 57% identical and 72% similar to the human homologue (zHook2; Fig. S5 E). The Hook domain is 65% identical and 78% similar between the two homologues. We first analyzed the localization of zHook2 cloned from cDNA of zebrafish embryo at 24 h post-fertilization (hpf). HA-tagged zHook2 localized to centrosomes marked by γ-tubulin staining (Fig. 9 I). Further, ectopic expression of zHook2 in HeLa cells dramatically relocalized endogenous p150^glued^ and DIC to the centrosomes (Fig. 9 I). Consistent with its ability to recruit dynein–dynactin, zHook2 partially rescued the dynein–dynactin interaction defect observed in Hook2-depleted cells, confirming an evolutionarily conserved role of Hook2 as a dynein–dynactin adaptor (Fig. 9 J and densitometric quantification shown in Fig. 9 K). We next investigated the phenotype of Hook2 depletion in zebrafish embryos. To this end, zHook2 was depleted in single cell embryos by morpholino microinjection. Due to the unavailability of antibodies to detect endogenous Hook2 in zebrafish embryos, an alternative approach involving transfection of control and zHook2 morpholino-treated HEK293T cells with either HA-tagged zHook2 or HA-tagged hHook2 was used for confirming the efficiency and specificity of morpholino treatment. It was observed that morpholino transfection reduced expression of only zHook2 but not hHook2 (Fig. S5 F). The morphants were observed for a period of 48 hpf where a dose-dependent mortality of zHook2 morphants was observed, suggesting an essential role of Hook2 in proper embryonic development of zebrafish (Fig. S5 G). Hook2 morphants injected with 0.25 mM and 0.5 mM concentrations of morpholino showed severe deformity of the body structures, such as defects in somites forming myotomes, defects in elongation of the body axis, and poorly defined head and tail buds (Fig. 9 L). These findings indicate that Hook2 is required for early development in zebrafish, likely functioning as a dynein–dynactin linker that regulates mitotic progression during embryonic cell division.

## Discussion

In this study, we have identified the mammalian Hook paralog Hook2 as a factor required for dynein–dynactin assembly, particularly during the onset of mitosis. Hook2 regulates dynein–dynactin localization at the NE and therefore, dynein function in anchoring of centrosomes to the NE. Independent of its binding to dynein, Hook2 regulates MT nucleation, leading to reduced astral MTs and defects in spindle positioning upon Hook2 depletion.

Hook2 binds to the LIC1 subunit of dynein via a highly conserved binding interface present in the Hook domain that was first identified in Hook3 (Schroeder and Vale, 2016). Point mutations in the LIC binding site of Hook2 abrogated binding to dynein, but not to dynactin (Fig. 1). These observations indicate that Hook2 (and possibly other Hook proteins) has distinct sites for binding to dynein and dynactin. Indeed, the cryo-EM structure of dynein tail-dynactin–Hook3 complex showed that the coiled-coil region of Hook3 runs along the length of the dynactin filament, and specifically, coiled-coil density from Hook3 (the identity of which is unclear) was observed near dynactin’s pointed end (Urnavicius et al., 2018). We also showed that Hook2, like the other Hook paralogs, was sufficient to activate dynein-based organelle motility, suggesting that Hook2 is also an activating dynein adaptor.

Dynein has multiple localizations and multiple functions in a mitotic cell, including centrosome anchoring to the NE, chromosome alignment, spindle pole focusing, spindle positioning, and spindle assembly checkpoint inactivation (Howell et al., 2001; Salina et al., 2002; Goshima et al., 2005; Varma et al., 2008; Raaijmakers et al., 2012, 2013). During the late G2 phase/early prophase of the cell cycle, dynein–dynactin localizes at the NE and mediates centrosome anchoring on the NE (Salina et al., 2002; Splinter et al., 2010; Raaijmakers et al., 2012, 2013). Accordingly, centrosome–NE distance is dramatically increased in dynein–dynactin-depleted cells (Raaijmakers et al., 2013; Fig. 3). Centrosome–NE anchoring is important for mitotic progression, as MTs nucleated by separating centrosomes exert pulling forces on the NE and facilitate the breakdown of the NE (Beaudouin et al., 2002; Mühlhäusser and Kutay, 2007). Two independent mechanisms of dynein recruitment at the NE have been proposed: (1) by BICD2 binding to Nucleoporin RanBP2/Nup358 (Splinter et al., 2010) and (2) by CENP-F–Nde1/L1 complex bound to nuclear pore complex protein Nup133 (Bolhy et al., 2011). Our findings suggest that Hook2 promotes dynactin and therefore dynein localization at the NE by mediating dynein–CENP-F interaction (Fig. 4). Accordingly, we noted a significant increase in centrosome–NE distance in Hook2-depleted cells that was rescued by WT but not dynein binding-defective Hook2 mutants. Notably, the *C. elegans* Hook homologue, ZYG-12, which localizes to centrosome as human Hook2 (Guthrie et al., 2009; Minn et al., 2009), also mediates centrosome attachment to the nucleus by regulating dynein localization at the NE (Malone et al., 2003). Supporting this idea of conservation of Hook2 function across evolution, we found that the zebrafish Hook2 homologue localized to the centrosomes, recruited dynein–dynactin subunits to the centrosome, and acted as a linker to promote dynein–dynactin interaction (Fig. 9).

Follow-up of mitotic progression in Hook2-depleted cells by live-cell imaging revealed a delay in chromosome congression and spindle positioning defects in these cells (Fig. 5). Both of these mitotic events, i.e., chromosome congression and spindle positioning, are regulated by dynein localized at the KT and cortex, respectively (Yang et al., 2007; Kiyomitsu and Cheeseman, 2012; Kotak and Gönczy, 2013; Amin et al., 2018). However, we did not find any significant change in KT and cortical dynein in Hook2-depleted cells (Fig. S3). Spindle positioning and KT-MT attachments (required for chromosome congression) are processes dependent upon spindle pole/centrosome-mediated MT nucleation (Heald and Khodjakov, 2015; di Pietro et al., 2016). Using two different approaches of measuring MT nucleation, i.e., MT regrowth over time after cold depolymerization and rate of emergence of newly nucleated MTs labeled by EB1-GFP, we conclude that Hook2 regulates MT nucleation from centrosomes (Fig. 6). The precise mechanisms underlying MT nucleation in a Hook2-dependent manner will require further detailed exploration, since the levels of centrosomal γ-tubulin, the major nucleating complex, remain relatively unperturbed (Fig. S3). Perhaps Hook2-dynein does not transport nucleating factors such as γ-tubulin to the centrosome, as is known for dynein function, but could localize to the centrosome independent of dynein and directly or indirectly modulate γ-TURC nucleation activity, for instance by regulating post-translational modification of γ-TURC complex (Haren et al., 2009; Teixidó-Travesa et al., 2012). We noted that Hook2-depleted cells also appeared to affect MT nucleation from noncentrosomal locations, such as around the chromosomes (Fig. 6 C; DAPI channel not shown). The potential role of Hook2 in acentrosomal MT nucleation, from the chromosomes in mitotic cells (Teixidó-Travesa et al., 2012) and from the Golgi complex in interphase (Sanders and Kaverina, 2015), where we observe Hook2 localization (Fig. S2 F), will also need further investigation. Nevertheless, the requirement of Hook2 for nucleating MTs from centrosomes is pronounced and provides a plausible explanation both for the chromosome congression defects (Auckland and McAinsh, 2015), as well as spindle mis-positioning/mis-orientation (Kiyomitsu and Cheeseman, 2012; Lu and Prehoda, 2013; McNally, 2013; di Pietro et al., 2016).

### Role of Hook2 during cytokinesis

In addition to centrosomes, we found Hook2 localization on the central spindles and midbody in anaphase and cytokinetic cells, respectively (Fig. 8). Here Hook2 was required for dynein–dynactin recruitment to the central spindles and dynactin-dependent targeting of the centralspindlin subunit MKLP1 to the midzone that was previously known in *Drosophila* S2 cells (Delcros et al., 2006; Fig. 8 and Fig. 9). Consistent with this study, we found a striking loss of MKLP1 from the midzone upon dynactin depletion in a small fraction of HeLa cells that escape metaphase arrest and enter anaphase (Fig. S4). Depletion of MKLP1 leads to delocalization of the RhoA GEF, ECT2, and centralspindlin subunit CYK4 from the central spindle, which broadens the region of RhoA activation leading to defects in cleavage furrow ingression (Yüce et al., 2005). Our findings suggest that Hook2, via its interaction with the dynein–dynactin complex, is required for localization of the centralspindlin complex at the midzone, giving a plausible mechanism for cytokinesis failure upon Hook2 depletion. Based on the approach used here (p150^glued^ siRNA), we cannot determine the mechanism underlying dynactin-dependent targeting of the centralspindlin complex to the midzone. To precisely understand the role of dynactin in midzone recruitment of centralspindlin subunits, it would be important in future studies to use targeted approaches of inhibiting dynactin function specifically at the central spindle. We also noted that Hook2 localized at the midbody ring in cytokinetic cells, wherein its function remains unexplored. It would be relevant to determine whether the role of Hook2 as a dynein–dynactin linker is required for endosomal trafficking within the midbody.

The ability of mitochondrially anchored Hook2 to induce robust centripetal transport of this organelle toward the peri-nuclear region supports a role for Hook2 as a dynein–dynactin adaptor (Fig. 1, J–L). Considering high sequence conservation of the “Hook domain” and Hook2 function in stabilizing the dynein–dynactin complex, it is highly probable that Hook2 (like Hook1 and Hook3) is also an activating adaptor that assists dynein–dynactin assembly into a processive motor complex. It would be important to confirm this hypothesis through in vitro motility assays and would also be interesting to determine, for instance by cryo-EM, whether all the three Hook paralogs induce a similar or distinct conformation of the dynein–dynactin complex. Another important question is to elucidate the precise mechanism by which Hook2 regulates MT nucleation. As centrosomal levels of γ-tubulin were unaffected upon Hook2 depletion, it is possible that Hook2 either directly or indirectly modulates γ-TURC nucleation activity. Future studies would provide new insights into Hook2 function and illuminate the regulatory mechanisms that govern its function as a dynein–dynactin adaptor.

## Materials and methods

### Cell culture

HeLa and HEK293T were procured from ATCC. Primary MEFs were isolated from 15-d-old BALB/c mice embryos and were provided by A. Tuli (Council of Scientific & Industrial Research [CSIR], Institute of Microbial Technology, Chandigarh, India). HeLa cells stably expressing GFP–α-tubulin and H2B-mCherry were a gift from D.W. Gerlich (Institute of Molecular Biotechnology, Vienna, Austria). All the cell lines were cultured in DMEM (Lonza) supplemented with 10% FBS (Gibco) in a humidified cell culture chamber with 5% CO_2_ at 37°C. Each cell line was screened continually for the absence of mycoplasma contamination using a MycoAlert Mycoplasma Detection Kit (Lonza) and was subcultured not more than 15 times.

### Synchronization of cell lines

For synchronization of the cell cycle, HeLa and HEK293T cells were arrested at the G1/S border by successive treatments with thymidine (2.5 mM) for 18 h with 8 h release in between. The second thymidine block was followed by release in S-phase, and cells were fixed or harvested at indicated stages of the cell cycle. Nocodazole (final concentration, 100 µM) was added to cells released from a second thymidine block to enrich cells in prometaphase (Fig. 2). In all cases where RNAi is performed in HeLa cells, the cell cycle synchronization was performed with a single thymidine block for 18 h followed by release for 8 h. RO-3306 (final concentration 9 µM; Fig. 4 A) and nocodazole (final concentration, 100 µM; Fig. 6 N and Fig. S3 A) were added 4 h after release from thymidine block to arrest cells in G2 phase and prometaphase, respectively. Primary MEFs were synchronized by serum starvation for 48 h followed by stimulation using complete media containing 20% FBS.

### siRNA treatment

The siRNA oligos for gene silencing studies were purchased from GE Healthcare (Dharmacon) and prepared according to the manufacturer’s instructions. siRNA oligos were transfected (final concentration, 250 nM) using Dharmafect-1 (GE Healthcare) according to the manufacturer’s instructions. Sequences of siRNA oligos used in the study are as follows: control siRNA, 5′-TGGTTTACATGTCGACTAA-3′; Hook1, 5′-GAATGAACGTATTGAGGAATT-3′; Hook2, 5′-GGAGACTCTGATTTTATATTT-3′ and ON-TARGET^Plus^ spool; Hook3, 5′-ACTGTCAGTCTAGAGGAAGAGTTTT-3′; mHook2, ON-TARGET^Plus^ spool; p150^glued^, 5′-GAAGATCGAGAGACAGTTATT-3′; and Dynein HC, 5′-GAGAGGAGGTTATGTTTAATT-3′.

### Mammalian/bacterial expression constructs

Full-length zebrafish Hook2 (Uniprot ID**:** A0A0R4IMZ5-1) was PCR amplified from cDNA prepared from 24 hpf zebrafish embryos and cloned with N-terminal HA-tag in pcDNA3.1(−) vector between *BamHI* and *NotI* restriction sites. All the bacterial and mammalian expression plasmids used in this study are listed in Table S1.

### Preparation of stable cell lines

H2B-mCherry, GFP–α-tubulin, EB1-GFP, and mCherry-UtrCH cloned in lentivector (pCDH-CMV-EF1-Hygromycin or pCDH-CMV-EF1-Puromycin) were transfected in HEK293T cells with lentiviral packaging plasmids for production of viral particles using X-tremeGENE HP DNA transfection reagent (Roche). Viral particles in culture supernatants were harvested 48 h after transfection and were concentrated using Lenti-X concentrator (Takara Bio). HeLa cells were infected with lentiviral particles in the presence of 8 µg/ml polybrene (Sigma-Aldrich). Transduced cells were selected under 300 µg/ml Hygromycin-B and 3 µg/ml Puromycin (Invitrogen). Expression of the transgenes was confirmed by fluorescence microscopy and flow cytometry.

### Antibodies and chemicals

The following antibodies were used in this study: rabbit anti-HA (H6908; Sigma-Aldrich), mouse-anti-MBP (E8038S; New England Biolabs), mouse anti-HA (MMS-101P; Covance), mouse anti-Myc (SC-40; Santa Cruz Biotechnology), mouse anti-α-tubulin (DM1α) clone (T9026; Sigma-Aldrich), mouse anti-p150^glued^ (610474; BD Biosciences), mouse anti-p50 (611002; BD Biosciences), mouse anti-dynein intermediate chain (904901; Biolegend), rabbit anti-Hook1 (ab151756; Abcam), rabbit anti-Hook2 (Ab no. 2; ab154109; Abcam), mouse anti-Hook3 (SC-398924; Santa Cruz Biotechnology), rabbit anti-γ-tubulin (T3320; Sigma-Aldrich); rabbit anti-MKLP1 (SC-867; Santa Cruz Biotechnology), rabbit anti–Aurora B (ab2254; Abcam), human anti-CREST (15–234; Antibodies), rabbit anti-Mad1 (PA5-28185; Thermo Fisher Scientific), rabbit anti-KIF4A (A301-074A; Bethyl Laboratories), rabbit anti-MgcRac–GTPase-activating protein/CYK4 (A302-797A; Bethyl Laboratories), rabbit anti-Arp1 (ab11009; Abcam), mouse anti-Hec1/Ndc80 (9G3; ab3613; Abcam), rabbit anti-Cyclin-B (Y106; ab32053; Abcam), rabbit anti-CENP-F (ab5; Abcam), rabbit anti-p50/dynamitin (ab133492; Abcam), and rabbit anti-PRC1 (EP1513Y; ab51248; Abcam). Rabbit anti-Hook2 (Ab no. 1) raised against C terminus (428–719 aa) of human Hook2 was a gift from H. Kramer (University of Texas Southwestern Medical Center, Dallas, TX). All the Alexa Fluor–conjugated secondary antibodies and DAPI were purchased from Thermo Fisher Scientific. HRP-conjugated goat anti-mouse and goat anti-rabbit antibodies were purchased from Jackson ImmunoResearch Laboratories. Thymidine, Cdk1 inhibitor/RO-3306, phalloidin, taxol, cycloheximide, and nocodazole were purchased from Sigma-Aldrich. The Eg5 inhibitor/STLC and Polo-like kinase 1 inhibitor/BI-2536 were purchased from Cayman Chemicals.

### Protein purification, pulldown assays, and immunoblotting

For recombinant protein purification, bacterial expression vectors encoding for GST or GST-tagged proteins were transformed into *Escherichia coli* Rosetta DE3 strain. Primary cultures of the transformed single colony were set up for 12 h at 37°C in Luria-Bertani (LB) broth containing 100 µg/ml ampicillin. Secondary cultures were set up in LB broth using 1% primary inoculum and incubated at 30°C for 5 h with 0.5 mM IPTG. After protein induction, bacterial cultures were centrifuged at 6,000×*g* for 10 min, washed once with PBS, and resuspended in buffer containing 20 mM Tris-HCl, pH 7.4, 150 mM NaCl, 1 mM EDTA, 0.5 mM DTT, 0.5% Triton X-100, and 5% glycerol with protease inhibitor tablet (Roche) and 1 mM PMSF (Sigma-Aldrich). Cells were lysed by sonication followed by centrifugal separation of inclusion bodies at 16,000×*g* for 15 min at 4°C. The supernatants were incubated with glutathione resin (G-Biosciences) for 2 h at 4°C to allow binding of GST and GST-tagged proteins, followed by extensive washing of beads with wash buffer (20 mM Tris-HCl, pH 7.4, 150 mM NaCl, 1 mM EDTA, 0.5 mM DTT, 0.5% Triton X-100, and 5% glycerol). Similarly, secondary cultures of MBP and MBP-tagged protein constructs transformed in *E. coli* Rosetta DE3 were induced at 16°C for 12 h with 0.2 mM IPTG in LB broth. Bacterial cultures were centrifuged, and cells were resuspended in column buffer (20 mM Tris-HCl, pH 7.4, 200 mM NaCl, 1 mM EDTA, and 1 mM DTT) containing protease inhibitor (Roche) and 1 mM PMSF (Sigma-Aldrich). The supernatant obtained after sonication followed by centrifugation as described above was incubated with amylose resin (New England Biolabs) for 2 h for binding of MBP and MBP-tagged proteins followed by washing with 50 column volumes of column buffer. For purified protein interactions, MBP and MBP-tagged proteins were eluted from beads using column buffer containing 10 mM maltose.

For MBP pulldown assays, whole-cell lysate of HEK293T cells prepared in ice-cold lysis buffer (20 mM Hepes, pH 7.4, 350 mM NaCl, 1 mM DTT, 2 mM MgCl_2_, 0.1% Triton X-100, and 2 mg/ml BSA) was incubated with MBP or MBP-tagged proteins bound to amylose beads at 4°C for 3 h. Samples were washed four times with lysis buffer, eluted by boiling them in Laemmli buffer, and analyzed by immunoblotting. All dynein–dynactin pulldowns were repeated at least three times on separate days starting from preparation of fresh whole-cell lysate.

Hook2-LIC1 purified protein interaction assays were performed as described previously (Schroeder and Vale, 2016) with minor modifications: 2 µg of MBP and MBP-tagged Hook2 fragments was incubated with equal amounts of either GST or GST-LIC1 (389–523 aa) bound to glutathione beads for 2 h in 250 µl of buffer (50 mM Tris, pH 7.4, 100 mM NaCl, 1 mM DTT, 0.1% Tween 20, and 2 mg/ml BSA). The resin was washed, and protein complexes were eluted in Laemmli buffer followed by analysis by immunoblotting. In brief, protein samples separated on SDS-PAGE were transferred onto polyvinylidene fluoride membranes (Bio-Rad Laboratories). Membranes were blocked overnight at 4°C in blocking solution (10% skim milk prepared in 0.05% PBS–Tween 20). Primary and secondary antibodies as mentioned were prepared in 0.05% PBS–Tween 20. The membranes were washed thrice for 10 min with 0.05% PBS–Tween 20 and 0.3% PBS–Tween 20 after a 2-h incubation with primary antibody and a 1-h incubation with secondary antibody, respectively. The blots were developed using a chemiluminescence-based method (Pierce).

### CoIP

HEK293T cells either synchronized to specific cell cycle stage or asynchronous were transfected with indicated siRNA duplexes. Lysates from HEK293T cells at respective cell cycle stages were treated with indicated siRNA and prepared in TAP lysis buffer (20 mM Tris-HCl, pH 7.4, 150 mM NaCl, 0.5% NP-40, 1 mM Na_3_VO_4_, 1 mM NaF, 1 mM PMSF, and protease inhibitor cocktail). The lysates were incubated with p rotein-A/G beads (Thermo Fisher Scientific) bound to indicated proteins for 3 h at 4°C followed by four washes in TAP wash buffer (20 mM Tris-HCl, pH 7.4, 150 mM NaCl, 0.1% NP-40, 1 mM Na_3_VO_4_, 1 mM NaF, and 1 mM PMSF). The samples were then separated on 8% SDS-PAGE followed by immunoblotting for further analysis. The results obtained were quantified by densitometry in ImageJ software. All the coIP experiments were repeated at least twice on separate days starting from preparation of fresh whole-cell lysates.

### Midbody isolation

Isolation of midbodies was performed as described previously (Skop et al., 2004). Briefly, HeLa cells were plated in two 10-cm dishes and synchronized by single thymidine block (2.5 mM thymidine in complete media for 16 h) followed by the release in complete media for 4 h. Nocodazole (100 ng/ml) was added 4.5 h after release for another 4.5 h to arrest cells in prometaphase. The mitotic cells were dislodged by shake-off and collected by gentle centrifugation at 400×*g* for 1 min at room temperature. The pellet of mitotic cells obtained was washed once with fresh complete media and released in complete media for 1 h. After 1 h of incubation, phalloidin and taxol (final concentration, 5 µg/ml) were added to stabilize the spindles, and cell suspension was incubated for again 1 min followed by lysis in spindle isolation buffer (2 mM Pipes, 0.25% Triton X-100, and 20 µg/ml taxol) containing protease inhibitor cocktail (Sigma-Aldrich) at room temperature. The midbodies were pelleted from the suspension by centrifugation at 400×*g* for 20 min at room temperature, and the obtained pellet was resuspended in 100 µl of Laemmli buffer. The proteins in the supernatant were precipitated using 55% TCA and then resuspended in 100 µl Laemmli buffer. An equal volume of both the samples was loaded on SDS-PAGE, and different proteins were detected by immunoblotting.

### Transfections and immunofluorescence

HeLa cells and primary MEFs were grown on glass coverslips and transfected with indicated siRNA for 36 h. For overexpression, the zHook2 construct was transfected in HeLa using X-tremeGENE-HP DNA transfection reagent (Roche) for 16–18 h. Cells were fixed in methanol for 7 min at −20°C followed by three washes in PBS. Fixed cells were incubated in blocking solution (5% FBS in PHEM buffer; 60 mM Pipes, 10 mM EGTA, 25 mM Hepes, and 2 mM MgCl_2_, final pH 6.8) for 30 min at room temperature, followed by incubations with primary and Alexa Fluor–conjugated secondary antibodies for 60 min and 30 min, respectively, with three washes with PBS in between. All the dilutions of primary and secondary antibodies were prepared in PHEM buffer. Finally, coverslips were washed thrice with PBS and mounted on glass slides in Fluoromount-G (SouthernBiotech).

For visualizing dynein and dynactin at NE upon Hook2 depletion, HeLa cells were treated with indicated siRNA oligos and synchronized using a single thymidine block to the late G2 phase. Cells were preextracted with 0.2% Triton X-100 in PBS before fixation in 3.7% PFA, as described previously (Raaijmakers et al., 2013). For visualizing dynein at the cortex, cells preextracted with 0.2% Triton X-100 were fixed in methanol for 5 min at −20°C followed by three washes in PBS as described previously (Tuncay et al., 2015). Fixed cells were incubated with primary antibodies overnight and secondary antibodies for 30 min. Z-stacks with 0.31 µm inter-stack spacing were acquired on an LSM710 laser-scanning confocal microscope (Carl Zeiss) equipped with a Plan-Apochromat 63×/1.4 NA oil immersion objective and a high-resolution microscopy monochrome cooled camera, AxioCam MRm Rev. 3 FireWire (D; 1.4 megapixels, pixel size 6.45 × 6.45 µm) using ZEN2012 imaging software (Carl Zeiss). All images of control and gene-specific siRNA with different markers were captured at the same illumination and detection settings, ensuring no pixel saturation. For quantification, z-stack images were imported into ImageJ software and converted to maximum intensity projections for further analysis. The representative maximum intensity projection images in figures were imported into Adobe Photoshop CS6 and formatted to 300 dpi resolution. Intensity measurements were performed on acquired images in ImageJ after background subtraction.

### FKBP12-rapamycin-FRB binding assays

HeLa cells grown on glass coverslips were transfected with FRB-Fis1, DsRed2-Mito (for visualizing mitochondria), and FKBP12-tagged GFP or FKBP12-tagged GFP-Hook2 (WT/mutants) as indicated. Rapamycin (final concentration, 100 nM) was added for 2 h after 12 h of transfection, and cells were fixed in 4% PFA for 10 min. Single-plane confocal images corresponding to the maximum spread of transfected cells were acquired using an LSM710 confocal microscope. For quantifying mitochondrial distribution upon rapamycin-induced mitochondrial targeting of Hook2, the radii of the nucleus and the cell were determined using the eclipse selection tool of ImageJ. The region between the nucleus and the cortex of each cotransfected cell was divided into 10 concentric segments of equal thickness in ImageJ, and the DsRed intensity in each segment was calculated using ImageJ software and plotted as a function of relative distance from the nucleus.

### Morpholino microinjection in zebrafish embryos

For analysis of development, Hook2 morpholino (0.1–0.5 mM) was injected (∼5 nl) in single-cell zebrafish embryos (*n* = 100) using a Femtojet 4Xmicro-injector (Eppendorf AG). The injected embryos were observed for viability and development for 48 h. The number of dead embryos at indicated times was recorded, and dead embryos were discarded. Differential interference contrast images of developing embryos were acquired on an LSM710 laser-scanning confocal microscope equipped with an Achromat-Plan 10×/0.25 Ph1 objective and a high-resolution microscopy monochrome cooled camera, AxioCam using ZEN 2012 imaging software (Carl Zeiss). Translation blocker fluorescein-labeled morpholinos used for gene silencing study in zebrafish embryos were purchased from Gene Tools and prepared according to the manufacturer’s instructions. The sequences of morpholinos used in this study are as follows: control, 5′-CCTCTTACCTCAGTTACAATTTATA-3′ and zHook2, 5′-TGATGTTTATTCAAGCTCATGGTGC-3′.

### Live-cell time-lapse imaging

Time-lapse live-cell imaging was performed on HeLa cells stably expressing GFP–α-tubulin and H2B-mCherry or EB1-GFP and H2B-mCherry. Cells were grown on glass coverslips and transfected with indicated siRNA. Cells were imaged for 12 h after 36 h of siRNA transfection. For live-cell imaging, a customized aluminum slide containing 12-mm chambers was used as described previously (Mahale et al., 2016). Briefly, sterilized coverslips were used to seal one side of the chamber using VALAP (a 1:1:1 mixture of vaseline, lanolin, and paraffin). The conditioned media was filled in the well from the opposite side and was sealed with a coverslip containing adhered cells in 10% DMEM using sterile silicone grease. The time-lapse z-stack images of marked fields were acquired every 3 min for 12 h on a Leica TCS SP5II laser-scanning confocal microscope equipped with Leica DMi8 humidified heating incubator previously maintained at 37°C/5% CO_2_, HCX PL-APO CS 40×/63× 1.4 NA oil immersion objective, and Leica HyD using LAS-X imaging software. The Leica LAS X software was used to control various parameters during image acquisition as well as for image analysis.

For visualizing cytokinesis failure, HeLa cells stably expressing GFP–α-tubulin and mCherry-UtrCH were grown with complete media (10% DMEM) in 35-mm glass-bottom dishes (Eppendorf AG) and were treated with indicated siRNAs for 36 h. The cells in the metaphase were selected manually, and z-stack time-lapse images were acquired for 3 h every 3 min on an LSM710 laser-scanning confocal microscope equipped with a PeCon Insert P Set-2000 humidified heating incubator (PeCon) previously maintained at 37°C/5% CO_2_, Plan-Apochromat 40×/1.4 NA oil immersion objective, and high-resolution microscopy monochrome cooled camera AxioCam MRm Rev. 3 FireWire (D; 1.4 megapixels, pixel size 6.45 × 6.45 µm) using the ZEN2012 imaging software for real-time acquisition and postacquisition processing.

### MT regrowth assay

HeLa cells grown on glass coverslips and treated with indicated siRNA were synchronized with a single thymidine block for 16 h and released from the block when a majority of cells were in mitosis (9 h after release). Cells were shifted to 4°C by replacing the existing media with chilled complete media and kept on ice for 10 min. The media were again replaced with complete media at 37°C, transferred to 37°C, and fixed in methanol at indicated time points. The centrosomes were visualized with γ-tubulin, MTs with α-tubulin, and chromatin with DAPI. Z-stack images were acquired on an LSM710 confocal microscope, and MT length was calculated from 3D reconstructed images in the ZEN 2012 software.

### Spindle positioning and chromosome misalignment measurement

Spindle positioning in metaphase HeLa cells transfected with indicated siRNA was analyzed as function of angle between spindle pole axis and substratum as described previously (Zhu et al., 2013). To analyze the spindle orientation, HeLa cells plated on gelatin-coated coverslips were transfected with indicated siRNA for 36 h. Metaphase cells stained for α-tubulin and γ-tubulin were selected, and z-stack images (0.3 µm interval) were acquired by an LSM710 confocal microscope. The z-stack images were converted to volume view in ZEN Pro 2011 software (Carl Zeiss), and the spindle angle was determined by importing the volume view images in ImageJ software using the angle tool as shown in Fig. 5, J and K. The spindle pole axis was marked by joining the spindle poles (marked by γ-tubulin) with a straight line.

Chromosome misalignment was measured as described previously (Ali et al., 2017). Briefly, the distance of DNA spread was determined from maximum intensity projections of z-stack images of prometaphase/metaphase HeLa cells treated with indicated siRNAs by drawing a straight line parallel to the spindle pole axis in ImageJ software. The spindle pole axis was defined by joining the two centrosomes (stained with γ-tubulin) by a straight line in ImageJ.

### Measurement of dynein recruitment at the KTs

Dynein intensity at the KTs in nocodazole-arrested prometaphase HeLa cells treated with indicated siRNA or inhibitors was quantified as described previously (Etemad et al., 2015) with minor modifications. In brief, maximum intensity projections of z-stacks of prometaphase cells were projected using ImageJ macro. The regions of interest (ROIs) were marked using DAPI and Aurora B using the freehand selection tool in ImageJ, and the total intensity in red channel (dynein) was recorded. The same ROIs were also positioned manually in cytosol of the same cell to determine cytosol background, which was later subtracted from total intensity as described above to calculate dynein levels at the KTs. Similarly, the same ROIs were transferred to green channel (Aurora B) using ImageJ to calculate Aurora B levels at the KTs. The relative values of dynein to Aurora B intensity at the KTs were plotted in cells treated with indicated siRNAs.

### Measurement of dynein levels at the NE, cortex, and centrosome

Dynein intensity on the NE and cortex was quantified, as described previously (Tuncay et al., 2015). Briefly, maximum intensity projections of z-stacks of mitotic HeLa cells stained for dynein were generated in ImageJ. Rectangular ROI of 5 × 15 pixels was placed over five different positions on the cortex, and mean intensities from all the ROIs were recorded. The same ROI was transferred to cytosol, and mean cytosol intensity was calculated for each cell. The ratio of cortex to cytosol relative to control siRNA was reported. Similarly, the intensity of dynactin subunit p150^glued^ on the NE in the late G2 phase cells was calculated. For measuring dynein and dynactin on centrosomes, the intensity of dynein and dynactin on centrosomes in each siRNA/inhibitor treatment was determined from the maximum intensity projected images after background subtraction, and plotted as ratios relative to control siRNA treatment.

### Colocalization analysis

To analyze the presence of Hook2 at KTs, colocalization between Hook2 and dynactin subunit p150^glued^ was measured in each cell at both centrosomes and KTs. For measuring colocalization between Hook2 and p150^glued^ at KT, ROI was drawn over the image to mark KT using DAPI, and isolated by cropping out of whole image. Mander’s colocalization coefficient was determined after subtracting the cytosolic background in the JACOP plugin of ImageJ using threshold function in each case. For determining the colocalization at the centrosome, Mander’s colocalization coefficient between p150^glued^ and Hook2 was determined directly from the z-stack image after setting the threshold (45 and 65 in Hook2 and p150^glued^ channels, respectively) to quantify only the centrosomal signal. The threshold values for the respective channels were kept the same across all the images during analysis. Similarly, colocalization between DsRed-Mito and FKBP12-tagged-GFP-Hook2 (WT/mutants) was calculated from the images after background subtraction in ImageJ software.

### Measurement of the rate of MT nucleation

For analyzing the rate of MT nucleation, HeLa cells stably expressing EB1-GFP were treated with the indicated siRNAs and synchronized by a single thymidine block. The cells were released into fresh complete media (10% DMEM) for 7 h at 37°C, and z-stack time-lapse images were acquired every 5 s up to 5 min on a LSM710 laser-scanning confocal microscope equipped with a PeCon Insert P Set-2000 humidified heating incubator (PeCon) previously maintained at 37°C/5% CO_2_, Plan-Apochromat 63×/1.4 NA oil immersion objective, and a high-resolution microscopy monochrome cooled camera AxioCam MRm Rev. 3 FireWire (D; 1.4 megapixels, pixel size 6.45 × 6.45 µm) using ZEN2012 imaging software for real-time acquisition and postacquisition processing. The analysis of time-lapse z-stack images was performed as described previously (Salaycik et al., 2005) with minor changes. Briefly, to determine the rate of MT nucleation, the z-stack time-lapse image sequences were opened in Imaris 9 software (Bitplane), and the number of EB1-GFP growth events that originated from the centrosome was quantified for the entire duration of time-lapse videos and expressed as nucleations/unit time calculated using the spot detection and tracking plugin.

### Quantitative analysis of KT tension

HeLa cells grown on glass coverslips and treated with indicated siRNA were synchronized into metaphase by a single thymidine block for 16 h and released for 8 h where 10 µM MG132 was added after 6 h for the remaining 2 h to prevent anaphase onset. The cells were fixed in ice-cold methanol. The MTs were visualized by α-tubulin immunostaining, KTs with anti-Hec1 and anti-CREST antibodies, and chromatin by DAPI. Airyscan super-resolution z-stack images were acquired from 10–12 metaphase cells under identical settings on a LSM880 laser-scanning confocal microscope (Carl Zeiss) equipped with a Plan-Apochromat 100×/1.4 NA oil immersion objective and 32-bit GaAsP PMT Airyscan fast-module detector elements. Acquisition and 3D Airyscan processing of acquired images was done using ZEN 2.1 software (Carl Zeiss). The pixel size used for imaging was 20 nm with an image format of 2,048 × 2,048 pixels and 4× optical zoom. Inter-KT distance measurements were performed in ImageJ for 20–25 KT pairs per cell using the line tool. From the same images, the levels of KT proteins, Hec1 and CREST, were also determined. The intensity of Hec1 and CREST was determined, as previously described by Hoffman et al. (2001). Briefly, a circle of 1 µm diameter was centered over 15–20 KTs in each cell, and the intensity of each spot was calculated after background subtraction. The same ROIs were transferred to the CREST channel, and the intensity of the same spot in the channel was measured using ImageJ. The levels are reported after normalizing with the intensity of CREST at each KT.

### Quantitative analysis of the central spindle intensity

The quantification of dynein and dynactin localization at the central spindles was performed in anaphase cells treated with indicated siRNA as described previously (Maton et al., 2015). Briefly, maximum intensity projections of z-stacks of anaphase cells were projected using signal integration in ImageJ. For each image, 10 ROIs having XY size 100 × 30 pixels were positioned manually in the red-channel image within the region between the separated chromosomes (dynein intermediate chain or p150^glued^) using the line tool (30-pixel thickness). These 10 ROIs also included the 5 ROIs of same dimension randomly positioned in the cytosol within the same cell. The average red intensity in the central spindle region was calculated, and the mean cytosolic background was subtracted for each cell. The values of control and Hook2 siRNA-treated anaphase cells were plotted relative to mean central spindle intensity of control siRNA. For MKLP1, these ROIs were positioned on the spindle midzone and cytosol and processed in the similar manner as described above.

### Line scans along cytokinetic bridge

For quantitative analysis of cytokinetic bridge, 5-pixel-thick line scans were generated from maximum intensity projections of z-stacks of the late cytokinetic HeLa cells (transfected with indicated siRNA) using signal integration in ImageJ. The line scans over the cytokinetic bridges were positioned in such a way that the midbody region marked with MKLP1 or Aurora B lies in the middle of segment drawn. The average fluorescence intensities at different points along these scans were extracted and plotted with respect to the distance from the midbody.

### Statistical analysis

Statistical analysis and representation of the data were done in GraphPad Prism 7 software. Data are presented as mean ± SD, and the P values were calculated using two-tailed unpaired Student’s *t* test. The sample sizes (*n*) are specified in the figure legends for all of the quantitative data.

### Online supplemental material

Fig. S1 shows Hook2 interaction with dynein and dynactin, and overexpression of Hook2 enhances the interaction between the two complexes. Also, this figure shows that mitochondrial clustering in the perinuclear region upon Hook2 overexpression is dynein-dependent. Fig. S2 shows that Hook1 and Hook3 are degraded upon anaphase onset, in contrast to Hook2, which is not degraded during the analyzed time points. In addition, this figure also shows that endogenous Hook2 localizes to centrosome and Golgi. Fig. S3 shows that Hook2 depletion does not affect dynein localization to the centrosome (during G2 phase), KTs, and cortex during the cell cycle. Furthermore, this figure shows that recruitment of MT nucleating agents to the mitotic centrosomes is not affected upon Hook2 depletion, and Hook2 did not localize to KTs. Fig. S4 shows that Hook2 depletion results in early cytokinesis failure and also impairs dynactin localization at the cytokinetic bridge. In addition, this figure shows that the centralspindlin complex subunit MKLP1 failed to localize to the spindle midzone upon dynactin depletion. Fig. S5 shows that Hook2 but not Hook3 is required for the association between MKLP1 and dynactin. Furthermore, Hook2 depletion results in increased embryo mortality in zebrafish embryos. Video 1 is a time-lapse video showing the detachment of the centrosome from the nucleus upon Hook2 depletion. Videos 2, 3, and 4 show the mitotic progression in cells transfected with control siRNA, Hook2 siRNA, and Hook2 spool, respectively. Additionally, Videos 3 and 4 also show a mitotic delay in case of Hook2 depletion. Video 5 shows a reduction in the rate of MT nucleation from centrosomes during prophase upon Hook2 depletion. Video 6 shows Hook2 WT but not the truncation mutant (N612) rescues MT nucleation during prophase. Furthermore, this video also shows that the dynein binding-defective mutant of Hook2 rescues the rate of MT nucleation. Videos 7, 8, and 9 show the mitotic exit and cytokinesis progression in cells transfected with control siRNA, Hook2 siRNA, and Hook2 spool, respectively. Additionally, Videos 8 and 9 show early cytokinesis failure and formation of the binucleated cell upon Hook2 depletion. Video 10 shows the ingression followed by regression of cleavage furrow leading to early cytokinesis failure upon Hook2 depletion. The furrow, however, ingresses completely in control siRNA-treated cells. Table S1 details the plasmids used in this study.

## Supplementary Material

Supplemental Material (PDF)

Video 1

Video 2

Video 3

Video 4

Video 5

Video 6

Video 7

Video 8

Video 9

Video 10
