# Correction: The dynein adaptor Hook2 plays essential roles in mitotic progression and cytokinesis

**DOI:** 10.1083/jcb.20180418308222019c

**Published:** 2019-08-27

**Authors:** Devashish Dwivedi, Amrita Kumari, Siddhi Rathi, Sivaram V.S. Mylavarapu, Mahak Sharma

Vol. 218, No. 3, March 4, 2019. 10.1083/jcb.201804183.

The authors were recently alerted that some of the blot images used to generate Fig. 2 E were inaccurate and similar to images shown in Fig. 1 H, at different exposures. They contacted *JCB* to initiate a correction and sincerely apologize for the use of inaccurate images in Fig. 2 E due to errors during the figure preparation process. The authors provided images from existing experimental replicates for panel E to the *JCB* editorial office for assessment. The correction does not affect the conclusion of Fig. 2 E or any of the conclusions in the paper. The corrected version of Fig. 2 is shown here.

**Figure 2.**
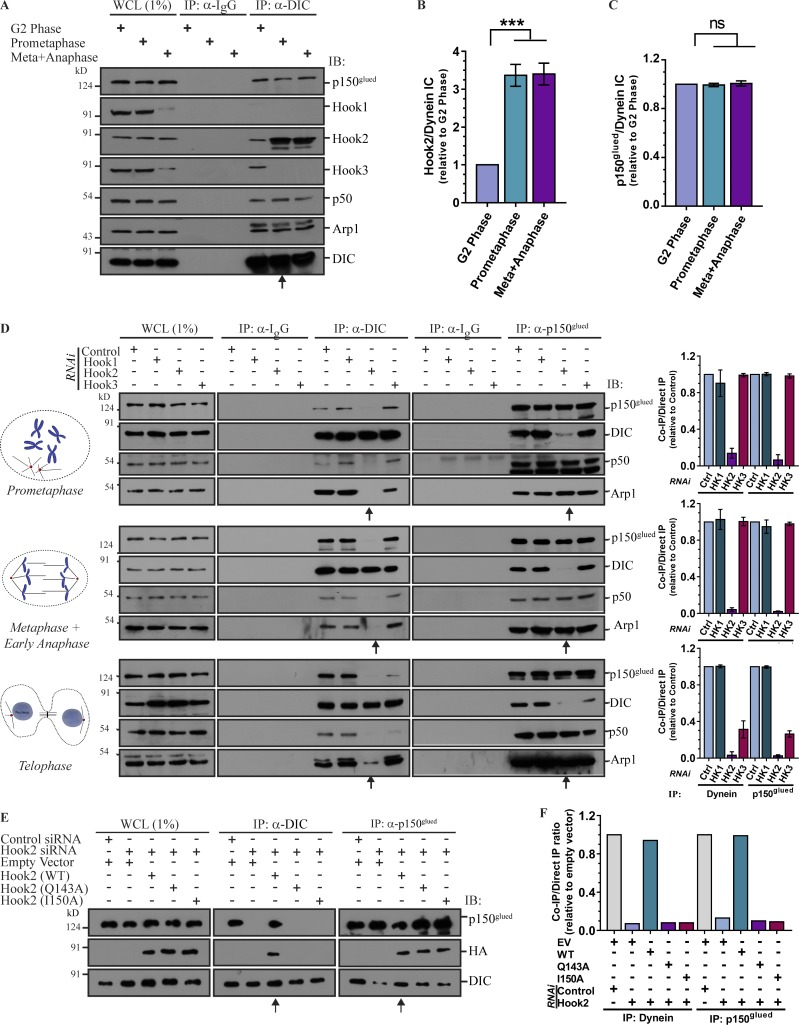
**Hook2 is required for dynein association with dynactin during mitosis. (A)** Lysates from HEK293T cells synchronized in G2 phase, prometaphase, and metaphase/anaphase were IP with control IgG or anti-DIC antibodies. The precipitates were IB with the indicated antibodies. Arrow mark prometaphase lane indicating an increased association of Hook2 with the dynein–dynactin complex at mitosis onset. **(B)** Ratio of normalized band intensity (G2 phase) of IP Hook2 to DIC in A (n = 4). **(C)** Ratio of normalized band intensity (G2 phase) of IP p150^glued^ to DIC in A (n = 4). **(D)** Lysates from HEK293T cells synchronized in prometaphase, metaphase/anaphase, and telophase and transfected with indicated siRNAs were IP with control IgG or antibodies against DIC or p150^glued^. The precipitates were IB with indicated antibodies. Arrows mark the lanes transfected with Hook2 siRNA. The bar graphs (on the right) represent normalized band intensity (control siRNA lane of respective cell cycle stage) of IP DIC to p150^glued^ and vice versa (n = 3). **(E)** Lysates from HEK293T cells synchronized in prometaphase and treated with control or Hook2 siRNA and transfected with EV or siRNA-resistant construct of Hook2 (WT/dynein binding-defective mutant) were tested for dynein–dynactin interaction as in D. Arrows mark the lanes transfected with Hook2 siRNA and siRNA-resistant Hook2 (WT). **(F)** Ratio of normalized band intensity (control siRNA) of IP DIC to p150^glued^ and vice versa in E (n = 2). Data represent mean ± SD (ns, not significant; ***, P < 0.001; Student’s t test).

In addition, it came to the authors’ attention that the pH3 panels shown in A and B of Fig. S2 looked similar to one another and that the tubulin panels shown in A and C of Fig. S5 looked similar to one another. The data in the paper were double checked by the authors and the authors provided original data as well as data from replicate experiments to the *JCB* editorial office for assessment. The panels shown in Fig. S2, A and B, and Fig. S5, A and C, were assembled correctly. No modification of those figures is needed.

The HTML and PDF versions of this article have been corrected. The error remains only in the print version.

