# Supplementary material for: The dynein adaptor Hook2 plays essential roles in mitotic progression and cytokinesis
Source: J Cell Biol. 2019 Mar 4;218(3):871–94. doi: 10.1083/jcb.201804183 (PMC6400558; doi:10.1083/jcb.201804183)
Supplement: Supplemental Material (PDF) [file JCB_201804183_sm.pdf]

Supplemental material

Dwivedi et al., https://doi.org/10.1083/jcb.201804183

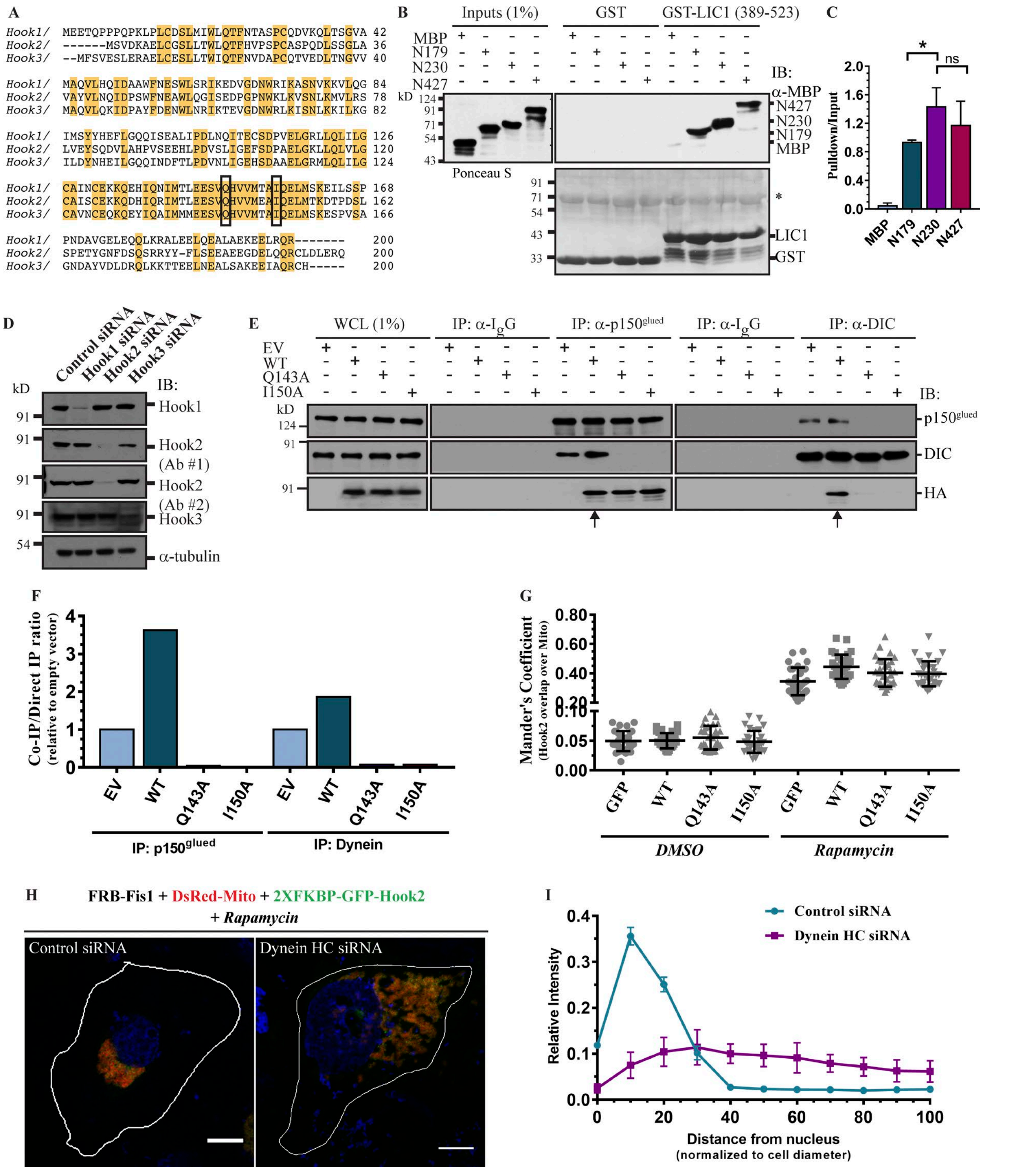

Figure S1. **Hook2 interacts with both dynein and dynactin. (A)** ClustalW alignment of Hook domains of human Hook paralogs indicating the positions of conserved residues (boxed) in all the paralogs crucial of LIC association. Yellow highlighted residues are conserved in all the Hook paralogs. **(B)** GST alone or GST-tagged LIC1 (389–523 aa) bound to glutathione beads were incubated with MBP-tagged fragments of Hook2, and IB with anti-MBP antibody for Hook2 fragments. LIC1 in the pelleted beads was detected using Ponceau S staining of the membrane. The asterisk indicates BSA protein band used for blocking glutathione beads. **(C)** Ratio of band intensity of pulldown to input Hook2 fragment signals in B (n = 3 independent experiments). **(D)** Whole-cell lysates from HeLa cells treated with indicated siRNA for 48 h were IB with anti-Hook protein antibodies, and α-tubulin was used as a loading control. **(E)** Lysates from HEK293T cells transfected with EV or Hook2-HA (WT or LIC binding-defective mutants) were incubated with protein-G beads bound to control IgG or antibodies against the DIC and p150^{glued} and centrifuged, and IP were IB for the indicated proteins. Arrows mark Hook2 (WT) transfected lanes. **(F)** Ratio of normalized band intensity (EV) of IP p150^{glued} to DIC and vice versa in E (n = 2). **(G)** Quantification of colocalization between GFP-Hook2 and DsRed2-Mito for images shown in Fig. 1 J (n = 3; 15–20 cells/experiment). **(H)** Representative images of FRB-FKBP12-rapamycin dimerization assay in fixed HeLa cells treated with indicated siRNAs. Bars, 10 µm. **(I)** Mitochondrial distribution in H quantified as intensity with respect to relative distance from the nucleus (n = 3; 10 cells/experiment). Data represent mean ± SD (ns, not significant; *, P < 0.1; Student's t test).

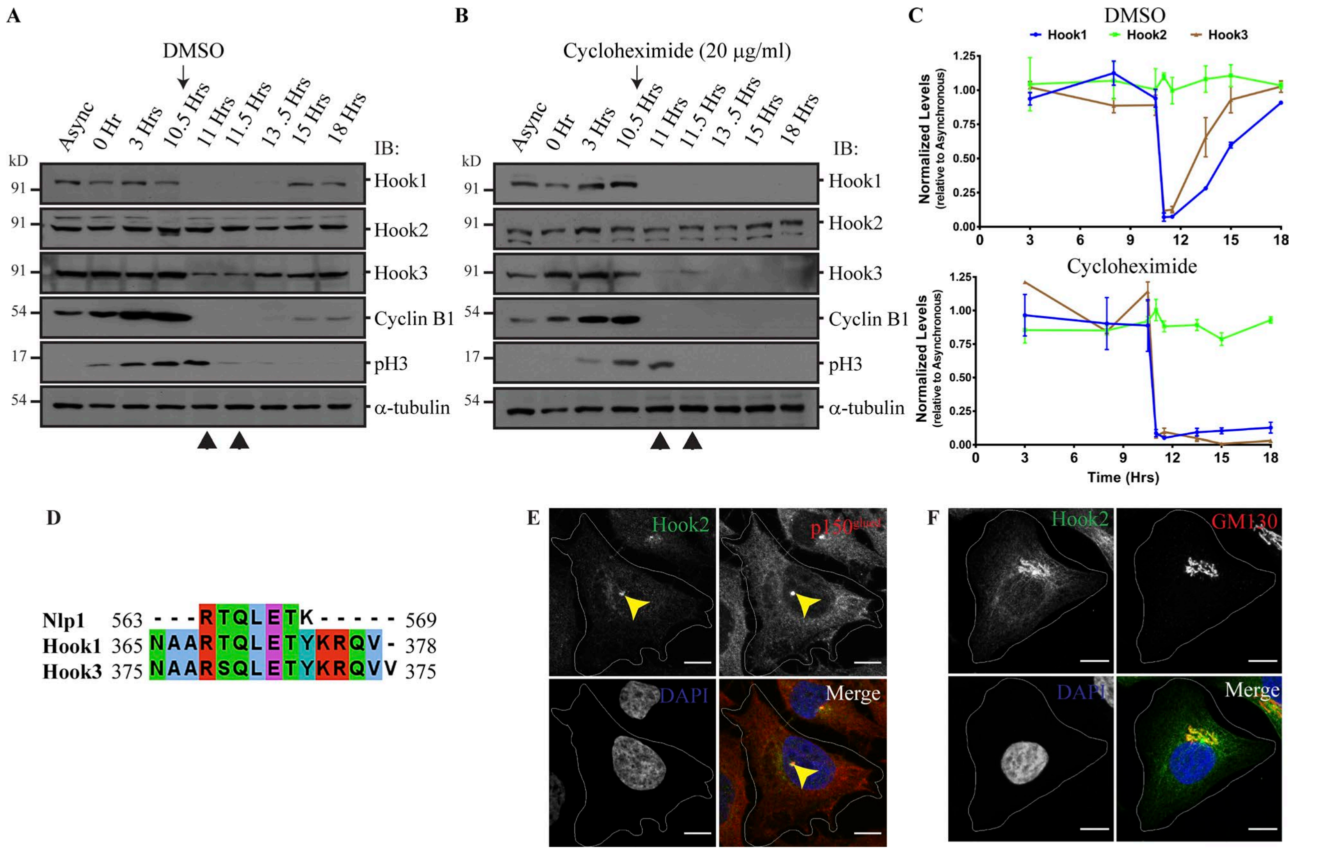

Figure S2. **Hook1 and Hook3 are degraded during mitosis. (A and B)** Lysates from HeLa cells synchronized by double-thymidine block to respective cell cycle stages, and IB for Hook proteins and α-tubulin. DMSO or cycloheximide was added at 10.5 h as indicated by the arrow. The arrowheads mark mitotic time points indicating degradation of Hook1 and Hook3 during mitosis. **(C)** Ratio of band intensity of Hook proteins in different cell cycle stages mentioned in A and B to band intensity of respective Hook paralogs in the asynchronous lane (n = 3). **(D)** ClustalW alignment of human Hook1 and Hook3 with D-box motif of Ninein-like protein-1 (Nlp1). **(E and F)** HeLa cells stained for endogenous Hook2; centrosomes were marked by p150^{glued}, and GM130 was used as a Golgi marker.

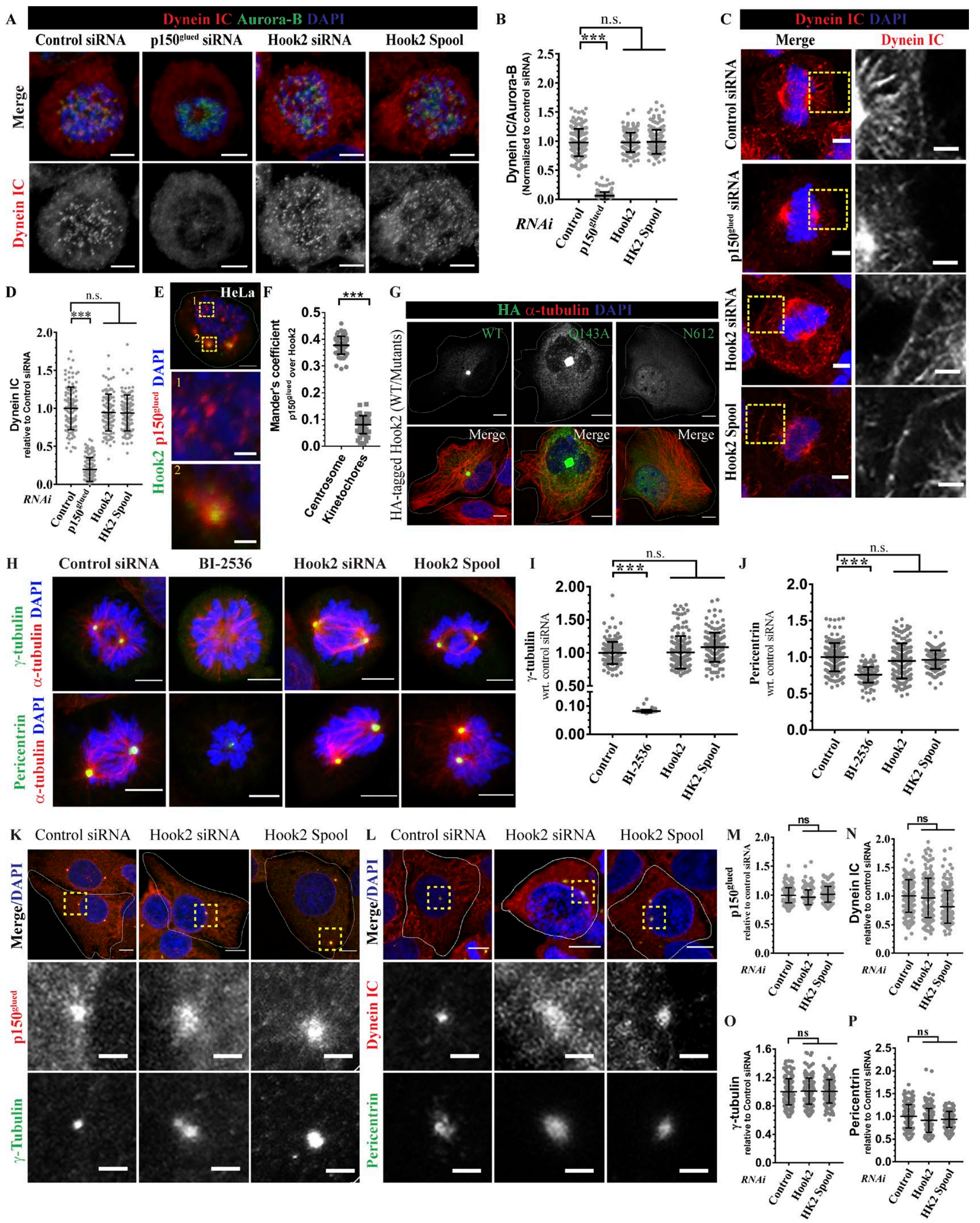

Figure S3. **Localization of dynein during different stages of cell cycle. (A)** Representative images of dynein at KTs in prometaphase-arrested HeLa cells treated that indicated siRNAs. KTs were stained with Aurora B and dynein with an anti-DIC antibody, and chromosomes were visualized with DAPI. Bars, 5 µm. **(B)** Quantification of the relative intensity of dynein at KTs (control siRNA) as described in A (*n* = 3; ∼50 prometaphase cells/experiment). **(C)** Representative images of dynein at the cortex in HeLa cells during metaphase upon indicated siRNA transfection. Dynein was stained with an anti-DIC antibody, and chromosomes were visualized with DAPI. Bars, 5 µm. **(D)** Quantification of the relative intensity of dynein at cortex (control siRNA) as described in C (*n* = 3; ∼20 metaphase cells/experiment). **(E)** Representative image of prometaphase-arrested HeLa cell stained for endogenous Hook2 and p150^{glued}. Bars, upper image, 5 µm; lower zoomed insets, 2 µm. **(F)** Quantification of endogenous Hook2 signal on the KTs measured using Mander's colocalization coefficient (p150^{glued} over Hook2) at the centrosomes and KTs (*n* = 2; 30 cells/experiment). **(G)** Representative image of HeLa cells transfected with indicated Hook2 (WT/mutants) constructs. Hook2 was stained with an anti-HA antibody and MTs with an anti-α-tubulin antibody, and the nucleus was visualized with DAPI. Bars, 10 µm. **(H)** Representative image of prometaphase HeLa cells transfected with indicated siRNAs. Centrosomes were marked by γ-tubulin and pericentrin and MTs by α-tubulin, and chromatin was visualized by DAPI. Bars, 5 µm. **(I and J)** Quantification of the levels of γ-tubulin and pericentrin shown in H (*n* = 3; 50 cells/experiment). **(K and L)** Representative images of HeLa cells during the G2 phase transfected with indicated siRNA, and stained for dynactin and dynein using antibodies for the p150^{glued} and DIC, respectively. Centrosomes were labeled with γ-tubulin and pericentrin. Bars, upper image; 10 µm; lower zoomed inset, 2 µm. **(M–P)** Quantification of levels of dynactin, dynein, γ-tubulin, and pericentrin during G2 phase described in K and L (*n* = 3; 50 cells/experiment). Data represent mean ± SD (ns, not significant; ***, P < 0.001; Student's *t* test).

https://doi.org/10.1083/jcb.201804183

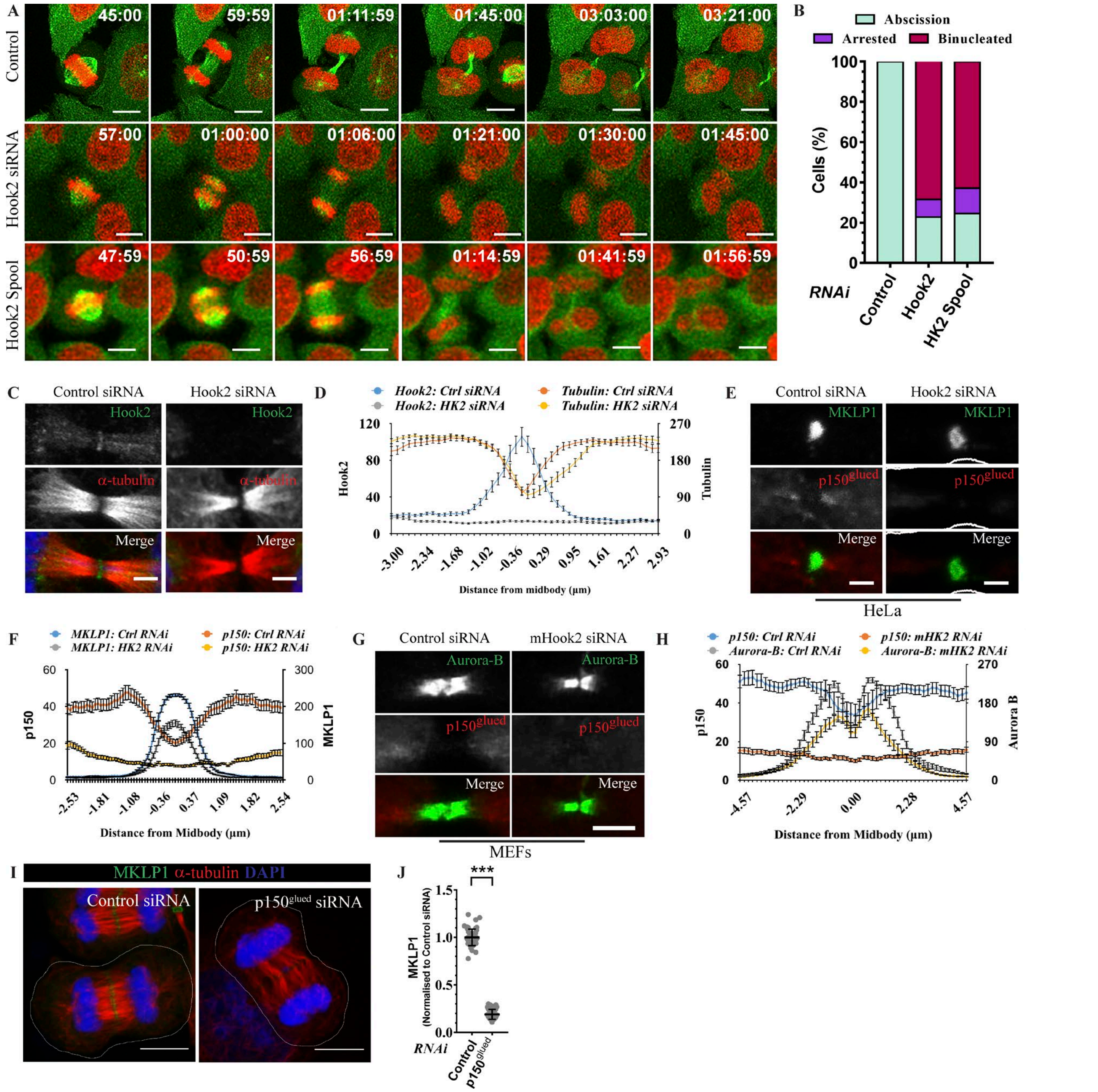

Figure S4. **Hook2 is required for localization of dynein and dynactin to the cytokinetic bridge. (A)** Maximum intensity projections of z-stacks of live-cell time-lapse imaging from HeLa cells stably expressing GFP–α-tubulin and H2B-mCherry transfected with indicated siRNA. Cells were imaged every 3 m to monitor mitotic progression in each case. Bars, 10 µm. **(B)** Quantification of the number of cells undergoing abscission or formation of binucleated cell in A from 70 mitotic cells. **(C)** Representative image of a cytokinetic bridge in HeLa cells treated with indicated siRNA, and stained for endogenous Hook2 and α-tubulin. Bars, 2 µm. **(D)** Quantification of the levels of Hook2 and α-tubulin in the cytokinetic bridge as described in A and measured by line scans (n = 3; 15 cells/experiment). **(E and F)** Representative image and line scan quantification of a cytokinetic bridge in HeLa cells treated with indicated siRNA and stained for p150^{glued}. Midbody was stained with MKLP1. Bars, 2 µm (n = 3; 15 cells/experiment). **(G and H)** Representative image and line scan quantification of a cytokinetic bridge in primary MEFs treated with indicated siRNA and stained for p150^{glued}. Midbody was stained with Aurora B. Bars, 2 µm (n = 3; 15 cells/experiment). **(I)** Representative image of HeLa cells in anaphase transfected with indicated siRNA. Spindles and midzone were stained with α-tubulin and MKLP1, respectively. Bars, 10 µm. **(J)** Quantification of the relative levels (control siRNA) of MKLP1 at spindle midzone in HeLa cells as described in I (n = 3; 15 cells/experiment). Data represent mean ± SD (***, P < 0.001; Student's t test).

https://doi.org/10.1083/jcb.201804183

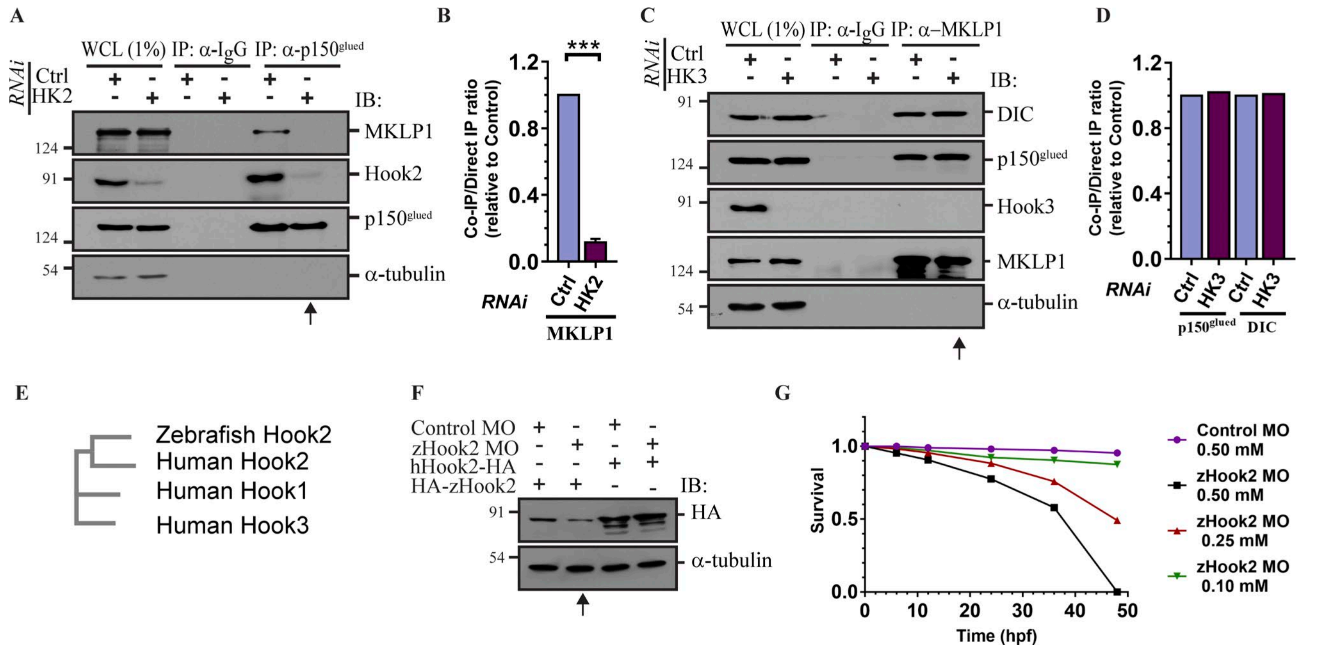

Figure S5. **p150glued mediates the interaction between Hook2 and MKLP1 during cytokinesis. (A)** Lysates of HEK293T cells synchronized by double-thymidine block, released and harvested at cytokinesis, and transfected with indicated siRNA were incubated with protein-G beads bound to an antibody against p150glued and centrifuged and IP were IB for the indicated proteins. Beads bound to control IgG antibody was used as a negative control in the experiment. **(B)** Ratio of band intensity of IP MKLP1 to p150glued in A normalized to control siRNA (*n* = 3). **(C)** Lysates of HEK293T cells transfected with indicated siRNA, synchronized by single-thymidine block, and harvested during cytokinesis were incubated with protein-A/G beads bound to an antibody against MKLP1 and centrifuged, and IP were IB for the indicated proteins. **(D)** Ratio of band intensity of IP p150glued and DIC to MKLP1 in C normalized to control siRNA (*n* = 2). **(E)** Cladogram showing the comparison of zebrafish Hook2 with human Hook paralogs. **(F)** Western blot analysis of lysates from HEK293T cells cotransfected with either HA-tagged zHook2 or hHook2 with either control morpholino or zHook2 morpholino to analyze the knockdown efficiency and specificity. **(G)** Survival of zebrafish embryos (*n* = 100) injected with either control (Ctrl) or indicated concentration of zHook2 morpholinos (MO) after fertilization.

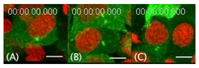

Video 1. **Visualization of detachment of centrosome with the nucleus in control versus Hook2 siRNA-treated HeLa cells.** Live-cell imaging of HeLa cells stably expressing EB1-GFP and H2B-mCherry and treated with control siRNA (A), Hook2 siRNA (B), and Hook2 spool (C). Every image was captured at an interval of 3 m immediately after the start of centrosome separation (total number of frames captured = 13). The video is shown at 2 frames per second (fps). Bars, 10 µm.

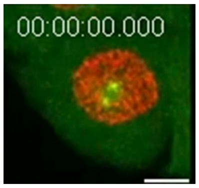

Video 2. **Visualization of the cell cycle in HeLa cells treated with control siRNA.** Live-cell imaging of control siRNA transfected HeLa cells stably expressing GFP–α-tubulin and H2B-mCherry with every image captured at an interval of 3 m immediately after NEBD (total number of frames captured = 60). The video is shown at 5 fps. Bar, 10 µm.

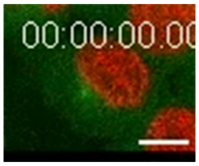

Video 3. **Visualization of the cell cycle in Hook2 siRNA-treated HeLa cells.** Live-cell imaging of Hook2 siRNA transfected HeLa cells stably expressing GFP–α-tubulin and H2B-mCherry with every image captured at an interval of 3 m immediately after NEBD (total number of frames captured = 53). The video is shown at 5 fps. Bar, 10 µm.

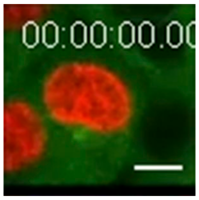

Video 4. **Visualization of the cell cycle in Hook2 spool siRNA-treated HeLa cells.** Live-cell imaging of Hook2 spool transfected HeLa cells stably expressing GFP–α-tubulin and H2B-mCherry with every image captured at an interval of 3 m immediately after NEBD (total number of frames captured = 47). The video is shown at 3 fps. Bar, 10 µm.

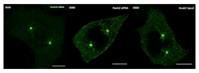

Video 5. **Visualization of the decrease in the rate of MT nucleation upon Hook2 depletion.** Live-cell imaging of indicated siRNA-treated prophase HeLa cells stably expressing EB1-GFP with every image captured at an interval of 5 s (total number of frames captured = 60). The video is shown at 4 fps. Bars, 10 µm.

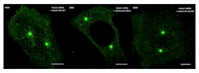

Video 6. **Visualization of rescue in the rate of MT nucleation upon ectopic expression of Hook2 localizing to centrosomes.** Live-cell imaging of prophase HeLa cells stably expressing EB1-GFP treated with Hook2 siRNA and transfected with indicated siRNA-resistant Hook2 construct. Every image was captured at an interval of 5 s (total number of frames captured = 60). The video is shown at 4 fps. Bars, 10 µm.

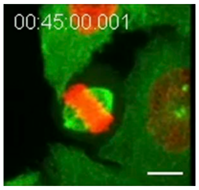

Video 7. **Visualization of the cytokinesis in control siRNA-treated HeLa cells.** Live-cell imaging of control siRNA transfected HeLa cells stably expressing GFP–α-tubulin and H2B-mCherry with every image captured at an interval of 3 m immediately after metaphase (total number of frames captured = 54). The time stamps shown in the video indicate duration after NEBD. The video is shown at 4 fps. Bar, 10 µm.

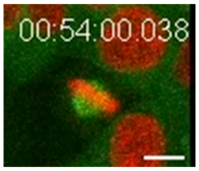

Video 8. **Visualization of the cytokinesis in Hook2 siRNA-treated HeLa cells.** Live-cell imaging of Hook2 siRNA transfected HeLa cells stably expressing GFP–α-tubulin and H2B-mCherry with every image captured at an interval of 3 m immediately after metaphase (total number of frames captured = 33). The time stamps shown in the video indicate duration after NEBD. The video is shown at 4 fps. Bar, 10 µm.

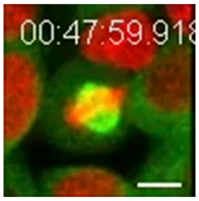

Video 9. **Visualization of the cytokinesis in Hook2 spool-treated HeLa cells.** Live-cell imaging of Hook2 spool transfected HeLa cells stably expressing GFP–α-tubulin and H2B-mCherry with every image captured at an interval of 3 m immediately after metaphase (total number of frames captured = 33). The time stamps shown in the video indicate duration after NEBD. The video is shown at 4 fps. Bar, 10 µm.

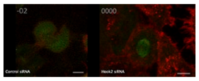

Video 10. **Visualization of the cytokinesis failure upon Hook2 depletion in HeLa cells.** Live-cell imaging of HeLa cells stably expressing GFP–α-tubulin and mCherry-UtrCH treated with indicated siRNA. Every image was captured at an interval of 3 m immediately after metaphase (total number of frames captured = 60). The video is shown at 4 fps. Bars, 10 µm.

Table S1. **List of molecular constructs used in this study**

Plasmid name	Description	Source
Bacterial expression constructs		
pMAL-C2X	Bacterial expression vector	New England Biolabs
pMAL-C2X-Hook2 (1–179)	Human Hook2 (1–179 aa) cloned in pMAL-C2X vector	This study
pMAL-C2X-Hook2 (1–230)	Human Hook2 (1–230 aa) cloned in pMAL-C2X vector	This study
pMAL-C2X-Hook2 WT (1–427)	Human Hook2 (1–427 aa) cloned in pMAL-C2X vector	This study
pMAL-C2X-Hook2 Q143A (1–427)	Human Hook2 (1–427 aa) with point mutation at amino acid position 143 changing Q with A cloned in pMAL-C2X vector	This study
pMAL-C2X-Hook2 I150A (1–427)	Human Hook2 (1–427 aa) with point mutation at amino acid position 150 changing I with A cloned in pMAL-C2X vector	This study
pGEX-4T3	Bacterial expression vector	GE Healthcare
pGEX-5X1-LIC1 (389–523)	Human LIC1 (389–523 aa) cloned into pGEX-5X1 vector	This study
Mammalian expression constructs		
pcDNA3.1(−)	Mammalian expression vector	Invitrogen
pcDNA3.1(−)-Hook2 (WT)-HA	Full-length human Hook2 with C-terminal HA-tag cloned in pcDNA3.1(−) vector	This study
pcDNA3.1(−)-Hook2 (Q143A)-HA	Full-length human Hook2 with C-terminal HA-tag and point mutation at amino acid position 143 changing Q with A cloned in pcDNA3.1(−) vector	This study
pcDNA3.1(−)-Hook2 (I150A)-HA	Full-length human Hook2 with C-terminal HA-tag and point mutation at amino acid position 150 changing I with A cloned in pcDNA3.1(−) vector	This study
pcDNA3.1(−)-HA-Hook2 N612	Human Hook2 (1–612 aa) with N-terminal HA-tag cloned in pcDNA3.1(−) vector	This study
pcDNA3.1(−)-2XFKBP12-GFP	2XFKBP12 with C-terminal GFP-tag cloned in to pcDNA3.1(−) vector	This study
pcDNA3.1(−)-2XFKBP12-GFP-Hook2 (WT)	Full-length human Hook2 with N-terminal tandem tag 2XFKBP-GFP cloned in pcDNA3.1(−) vector	This study
pcDNA3.1(−)-2XFKBP12-GFP-Hook2 (Q143A)	Full-length human Hook2 with point mutation at amino acid position 143 changing Q with A and N-terminal tandem tag 2XFKBP-GFP cloned in pcDNA3.1(−) vector	This study
pcDNA3.1(−)-2XFKBP12-GFP-Hook2 (I150A)	Full-length human Hook2 with point mutation at amino acid position 150 changing I with A and N-terminal tandem tag 2XFKBP-GFP cloned in pcDNA3.1(−) vector	This study
FRB-Fis1	Human Fis1 C-terminal tail (92-152) with N-terminal FRB tag cloned into pC_4-R_HE vector	Gift from Amit Tuli
DsRed2-Mito	Red fluorescent protein (DsRed2) and a mitochondrial targeting sequence of human cytochrome c oxidase subunit VII cloned into mammalian expression vector	Clontech
pcDNA3.1(−)-HA-zHook2	N-terminal HA-tagged full-length zebrafish Hook2 cloned in pcDNA3.1(−) vector	This study
pCDH-EF1-Hygro-H2B-mCherry	Human H2B with C-terminal mCherry-tag cloned in pCDH-CMV-MCS-EF1-Hygro vector	This study
pCDH-EF1-Puro-GFP–α-tubulin	Human α-tubulin with N-terminal GFP-tag cloned in pCDH-CMV-MCS-EF1-Puro vector	This study
pCDH-EF1-Puro-EB1-GFP	Full-length human EBI (MAPRE1) with C-terminal GFP-tag cloned into pCDH-CMV-MCS-EF1-Puro vector	This study
pCDH-EF1-Hygro-mCherry-UtrCH	Calphnin homology domain of human Utrophin (1–261 aa) with N-terminal mCherry tag cloned into pCDH-CMV-MCS-EF1-Hygro vector	This study

Constructs used as templates for cloning human Hook protein constructs were provided by Helmut Kramer (UT Southwestern Medical Center, Dallas, TX), for LIC1 by Takashi Muryama (Juntendo University School of Medicine, Tokyo, Japan), and for H2B-mCherry by Sachin Kotak (Indian Institute of Science, Bangalore, India).
